# Climate change impact on wheat and maize growth in Ethiopia: A multi-model uncertainty analysis

**DOI:** 10.1371/journal.pone.0262951

**Published:** 2022-01-21

**Authors:** Fasil Mequanint Rettie, Sebastian Gayler, Tobias K. D. Weber, Kindie Tesfaye, Thilo Streck

**Affiliations:** 1 Institute of Soil Science and Land Evaluation, Biogeophysics, University of Hohenheim, Stuttgart, Germany; 2 Ethiopian Institute of Agricultural Research, Melkasa, Ethiopia; 3 International Maize and Wheat Improvement Centre (CIMMYT), Addis Ababa, Ethiopia; Institute of Genetics and Developmental Biology Chinese Academy of Sciences, CHINA

## Abstract

Ethiopia’s economy is dominated by agriculture which is mainly rain-fed and subsistence. Climate change is expected to have an adverse impact particularly on crop production. Previous studies have shown large discrepancies in the magnitude and sometimes in the direction of the impact on crop production. We assessed the impact of climate change on growth and yield of maize and wheat in Ethiopia using a multi-crop model ensemble. The multi-model ensemble (n = 48) was set up using the agroecosystem modelling framework Expert-N. The framework is modular which facilitates combining different submodels for plant growth and soil processes. The multi-model ensemble was driven by climate change projections representing the mid of the century (2021–2050) from ten contrasting climate models downscaled to finer resolution. The contributions of different sources of uncertainty in crop yield prediction were quantified. The sensitivity of crop yield to elevated CO_2_, increased temperature, changes in precipitations and N fertilizer were also assessed. Our results indicate that grain yields were very sensitive to changes in [CO_2_], temperature and N fertilizer amounts where the responses were higher for wheat than maize. The response to change in precipitation was weak, which we attribute to the high water holding capacity of the soils due to high organic carbon contents at the study sites. This may provide the sufficient buffering capacity for extended time periods with low amounts of precipitation. Under the changing climate, wheat productivity will be a major challenge with a 36 to 40% reduction in grain yield by 2050 while the impact on maize was modest. A major part of the uncertainty in the projected impact could be attributed to differences in the crop growth models. A considerable fraction of the uncertainty could also be traced back to different soil water dynamics modeling approaches in the model ensemble, which is often ignored. Uncertainties varied among the studied crop species and cultivars as well. The study highlights significant impacts of climate change on wheat yield in Ethiopia whereby differences in crop growth models causes the large part of the uncertainties.

## Introduction

In large parts of the world, local economies and food security are dependent on agriculture which is very sensitive to climate variations [1,2]. Both shifts in the mean climate as well as changes in the variability can considerably affect agricultural production [3]. There is now insurmountable evidence linking anthropogenic greenhouse gas emissions to climate change [4]. If global warming continues at the current pace, the global average temperature is likely to increase by 1.5°C by 2050 compared to pre-industrial levels [3]. This will undermine agricultural production in many parts of the world, in general, and Ethiopia, in particular. It must be seen as a very severe threat to meeting the growing world food demand [5,6]. A higher and faster temperature increase of 2°C is projected for Africa [7]. This would lead in a major food security risk by 2050 [8]. Therefore, adapting agriculture to the changing climate is an additional and daunting problem for the continent [9]. Consequently, climate change impact assessment has developed as an important area of research in Africa [6,10], aiming at identifying adaptation options.

The impact of climate change on crop production has been studied extensively using statistical models [5,11,12], process-based crop models [13–15], and a suitability evaluation approach [16]. Simulations with process-based crop models driven by climate projections from dynamical downscaling have dominated the recent literature. Crop models can describe crop responses to different climate scenarios and variable agronomic management options. Globally, crop model structures exhibit quite a large variability enabling more robust projections and quantifying associated model uncertainties. This will improve the relevance of crop model outputs in different environments [17]. However, not accounting for the related uncertainties may jeopardize decision-making in the agricultural sector. There are many sources of uncertainties including those related to differences in the governing process model equations and parameterizations, climate model projections, and methods of climate downscaling. In order to help decision makers to decide between plausible decisions options, Challinor et al. [18] recommended that these sources of uncertainties should be distinguished and communicated.

Considering the complex and non-linear influence of climate on crop development and growth, quantifying, and dissecting the various sources of uncertainties in the prediction of the impact of climate change is a challenge. Recently, the multi-model ensemble approach has been introduced to study uncertainties in projected climate change impact on crop production [14,19,20]. By this, yield projections could be improved by calculating the ensemble means and quantifying the related uncertainties in comparison to the common single crop model-single climate projection simulations. One major finding from the previous studies was that differences in the crop models were the main source of uncertainty and usually dominated over the uncertainties resulting from the climate model scenarios [14,17,20].

Crop models differ not only in the equations describing crop growth, but also in the level of detail of boundary conditions needed for running them, and the equations representing related processes in the soil [21]. The role of different approaches in modelling these processes determining crop growth has so far not been analyzed in previous studies. For instance, modeling soil processes is an important component of crop modeling. However, considerable uncertainties remain to be addressed regarding the quality of predictions among different soil models of despite their significant progresses in soil models [22]. In previous multi-model studies such as that by Asseng et al. [23], the participating modeler groups contributed only with complete models, i.e. with a given combination of model equations that describe the soil-plant-atmosphere system. Ceteris paribus analyses of how single model components affect the outcome of simulations were not performed, which limits assigning uncertainties to individual processes.

Studies focusing on the influence of climatic variations on crop productivity are of particular importance for regions such as Ethiopia, where only few studies have been conducted so far. Ethiopia’s national economy is dominated by the agricultural sector, which accounts to 42% of the GDP, employs 85% of the population, and contributes about 90% to total export earnings [24]. Crop production is the main agricultural activity, and it is mainly practiced under rain-fed conditions. This can exacerbate the negative effects of climatic variations [25]. Since cereals are the main source of food, failures in cereal production will almost inevitably jeopardize food security. In 2018, 87% of total crop production were cereals grown on 80% of the total cultivated land [24]. Of the many cereals grown in Ethiopia, maize (*Zea mays* L.) and wheat (*Triticum aestivum* L.) are two of the three most important crops with a combined share of 42.6% of the total crop production [24].

Significant increasing trends in frequencies and magnitude of climate extremes have been documented in Ethiopia [26–28]. In the face of a changing climate, the likely increase of extreme events will have an adverse impact on cereal crop production in Ethiopia [10,29,30]. However, the results have shown large discrepancies in the magnitude and sometimes in the direction of the impact. For example, some studies projected maize yield to decline by 20–43% [31–33], other studies show that maize yield could increase by 2–51% [10,29,31]. The possible reasons of these inconsistencies might stem from limitations related to either the low number of crop models used, or the few numbers of climate model projections considered in each of the studies, or both.

Thus, the main objectives of this study were i) to quantify the impact of projected climate change on maize and wheat yields in Ethiopia, and ii) to dissect the contributions of different sources of uncertainties from the overall uncertainty in crop yield projections. To achieve the objectives, we applied a multi-model ensemble approach by running 48 crop growth and soil submodel combinations in conjunction with climate projections of ten different climate models from the Coordinated Regional Climate Downscaling Experiment (CORDEX). To the authors’ best knowledge, no crop modeling study has been conducted so far using dynamically downscaled climate projections in East Africa in general and Ethiopia in particular. Models were calibrated for the phenology and growth across 3 locations representing the major wheat and maize growing areas in Ethiopia. To identify the most influential factors that affect grain yield, a sensitivity analysis was also conducted by discretely varying selected input variables and analyzing their sensitivity on modeled yield for the 30 baseline years (1981–2010). The potential impact of climate change on yield was computed by comparing the relative changes between the baseline and future climate simulations. Finally, comparison was made for the contribution of the different sources of uncertainty in projected impact on grain yield.

## Materials and methods

### The study sites

**Fig 1:**
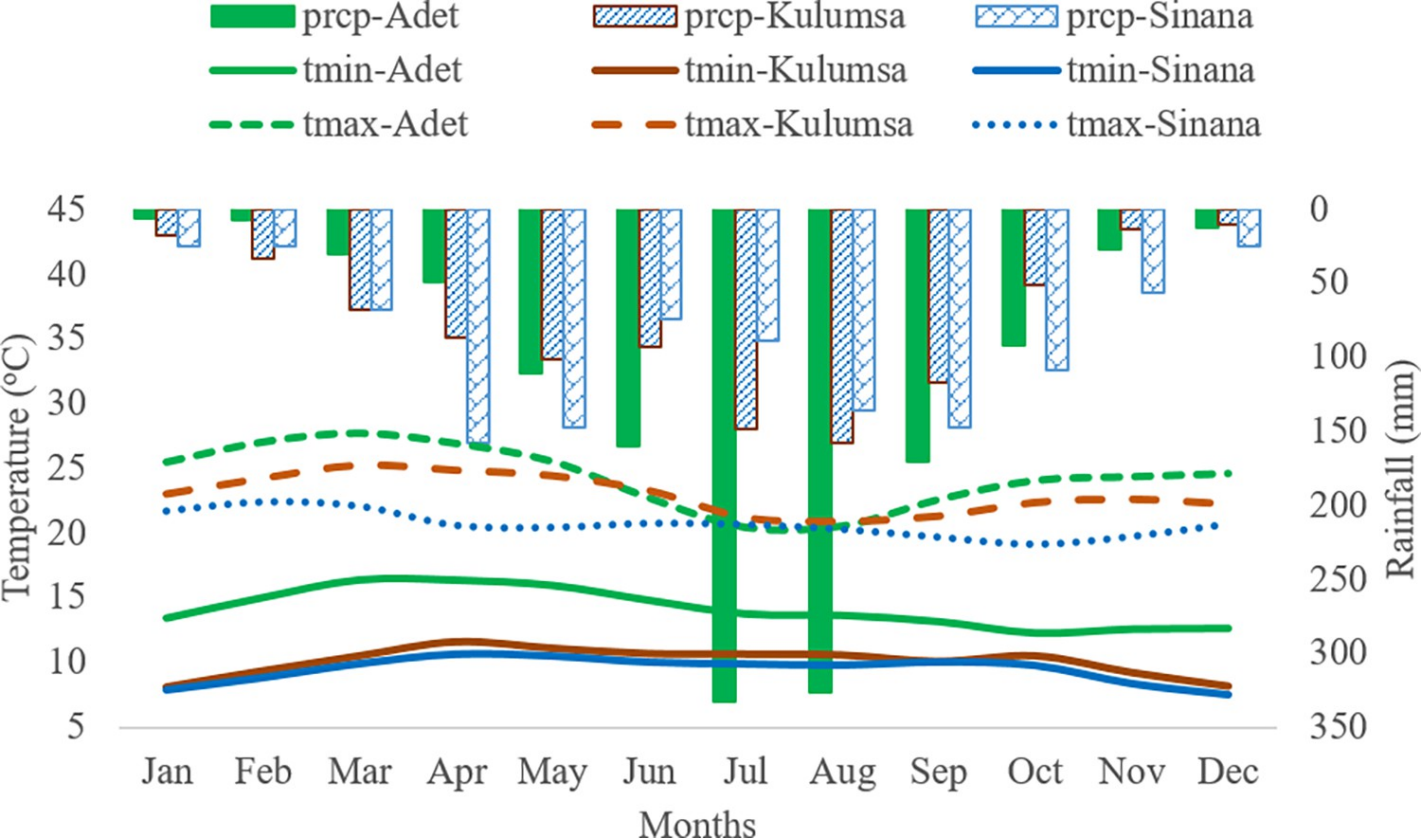
Monthly means of observed rainfall (prcp), and maximum (tmax) and minimum (tmin) temperature for 1981–2010 at the three research sites.

Our study was based on experimental field data for two wheat and two maize cultivars, which were collected over several years at three research sites with differing soil and climate: Adet and Sinana for the two wheat cultivars and Kulumsa for the two maize cultivars. Adet lies at an altitude of 2170 m with a long-term (average) annual rainfall between 1000 and 1800 mm (Fig 1). The dominant soil types in Adet are Fluvisols and Vertisols. Wheat is widely grown as the major crop following teff. Sinana is one of the major wheat producing regions in the country. It is located at the foot of the Bale Mountain west of the Great Rift Valley at an altitude of 2460 m. Its average annual rainfall varies from 600–1600 mm with two distinct rainy seasons; the main one from July-October and the minor one from March-June. The research site is characterized by a deep, fine textured and aggregated soil structure. Predominant soil types are Phaeozems and Cambisols. Kulumsa is in the central part of the country at an altitude of 2260 m. It represents a highland area with a cool climate receiving an annual total average rainfall of 700–1100 mm rainfall and a rainy season from May to October. Most of the area is a flat plain dominant by Haplic Xerosol.

### Observed climate and crop data

Observed daily weather data (rainfall, solar radiation, maximum temperature, and minimum temperature) were obtained from the National Meteorological Agency of Ethiopia for the period of 1981 to 2017. Datasets from the Agriculture Modern-Era Retrospective Analysis for Research and Applications [34] were used both for gap-filling and as source to estimate unavailable data such as wind speed and relative humidity. Field data for wheat was obtained from Adet and Sinana agricultural research centers while the data for maize was obtained from field experiments conducted by the International Maize and Wheat Improvement Center (CIMMYT) in collaboration with Kulumsa agricultural research center. The experimental data we used were gathered in parts during genotype by environment experiments and extracted from the agricultural research centers’ field books. The records contain summary of measured data, published in annual reports for each season and stored in the research centers’ libraries. The reports include records of crop development and growth observation data as well as soil and crop management information for each field experiment. Crop data include maturity date, anthesis date and grain yield, whereas crop management data include planting date, planting density and fertilizer application dates and rates. The reports include only the average values across the replications of the experiment. The four crop cultivars considered in the study were ‘Shina’ (in Adet) and ‘Medawolabu’ (in Sinana) for wheat as well as ‘Jibat’ and ‘Wenchi’ (in Kulumsa) for maize (Table 1). Available soil data included soil texture as well as physical and chemical properties (Table 2). The soil data were extracted from the report that match the beginning of our experimental crop data wherever available. For maize (i.e., Kulumsa site), soil data from the 2012 report were used whereas for wheat the data were extracted from the 2002 (i.e., Adet site) and 2006 (i.e., Sinana site) report.

**Table 1:** Description of crop cultivars used in the study.

Crop	Cultivar name	Year of release	Maturity group	On-station (on-farm) yield (t ha^-1^)	Days to maturity	Data collected (years)
Maize	Wenchi	2008	Intermediate	8–12 (6–8)	160–200	2010-2012/2015-2017
Maize	Jibat	2009	Intermediate	8–12 (6–8)	160–200	2011–2016
Wheat	Shina	1999	Intermediate	3–5 (2.5–3.5)	100–120	2001–2006
Wheat	Medawolabu	2000	Late	4–5 (3–4)	136–143	2006–2011

**Table 2:** Soil physical and chemical characteristics of soil horizons at the research sites.

Site	Depth (cm)	Silt (%)	Clay (%)	BD (g cm^-3^)	pH	OC (%)	PWP (Vol.%)	FC (Vol.%)	CEC (cmolc kg^-1^)
Adet	0–5	31	40	1.02	8.7	4.17	31.5	44.3	37
	5–15	31	41	1.07	8.7	4.1	31.5	43.5	37
	15–30	31	44	1.16	8.7	2.5	31.5	42.3	37
	30–60	28	46	1.20	8.7	1.9	31.8	41.8	36
	60–100	28	47	1.24	8.7	1.5	31.5	40.8	36
	100–125	27	47	1.26	8.7	1.0	31.5	40.5	38
Kulumsa	0–5	28	42	1.21	5.3	2.6	31.0	41.0	41
	5–15	28	44	1.23	5.3	2.4	31.0	41.0	40
	15–30	28	46	1.35	5.3	1.4	31.0	39.0	40
	30–60	25	47	1.40	5.3	1.0	31.0	38.2	41
	60–100	25	47	1.45	5.3	0.6	30.7	37.7	40
	100–150	26	45	1.47	5.3	0.5	30.0	37.0	41
Sinana	0–5	26	39	1.12	6.7	3.3	31.5	42.5	45
	5–15	26	41	1.18	6.7	2.9	32.0	42.0	46
	15–30	25	45	1.26	6.7	2.1	32.0	41.0	46
	30–60	24	47	1.30	6.7	1.6	32.5	40.5	47
	60–100	24	47	1.39	6.7	1.4	32.0	39.0	46
	100–145	24	47	1.45	6.7	1.2	31.5	37.5	45

BD: Bulk density; OC: Organic carbon; PWP: Permanent wilting point; FC: Filed capacity; CEC: Cation exchange capacity.

### Climate projections

Future climate change data of ten contrasting Global Climate Models (GCMs, S1 Table) were accessed from CORDEX [35] which had been downscaled to 44 km horizontal resolution in Africa (available at https://esgf-node.llnl.gov/search/esgf-llnl/). Sponsored by the World Climate Research Program, CORDEX generated high-resolution future climate projections by downscaling GCM projections using different regional climate models. A 30-year window period representing climate projections by the mid of the century (2021–2050) was considered in this study. A single Representative Concentration Pathway (RCP4.5) emission scenario was selected as the radiative forcing does not diverge in the different RCPs by the selected time [36]. The data were bias-corrected using the quantile-quantile mapping procedure [37,38], in order to match distributional characteristics of historical observations of precipitation and temperature.

**Fig 2:**
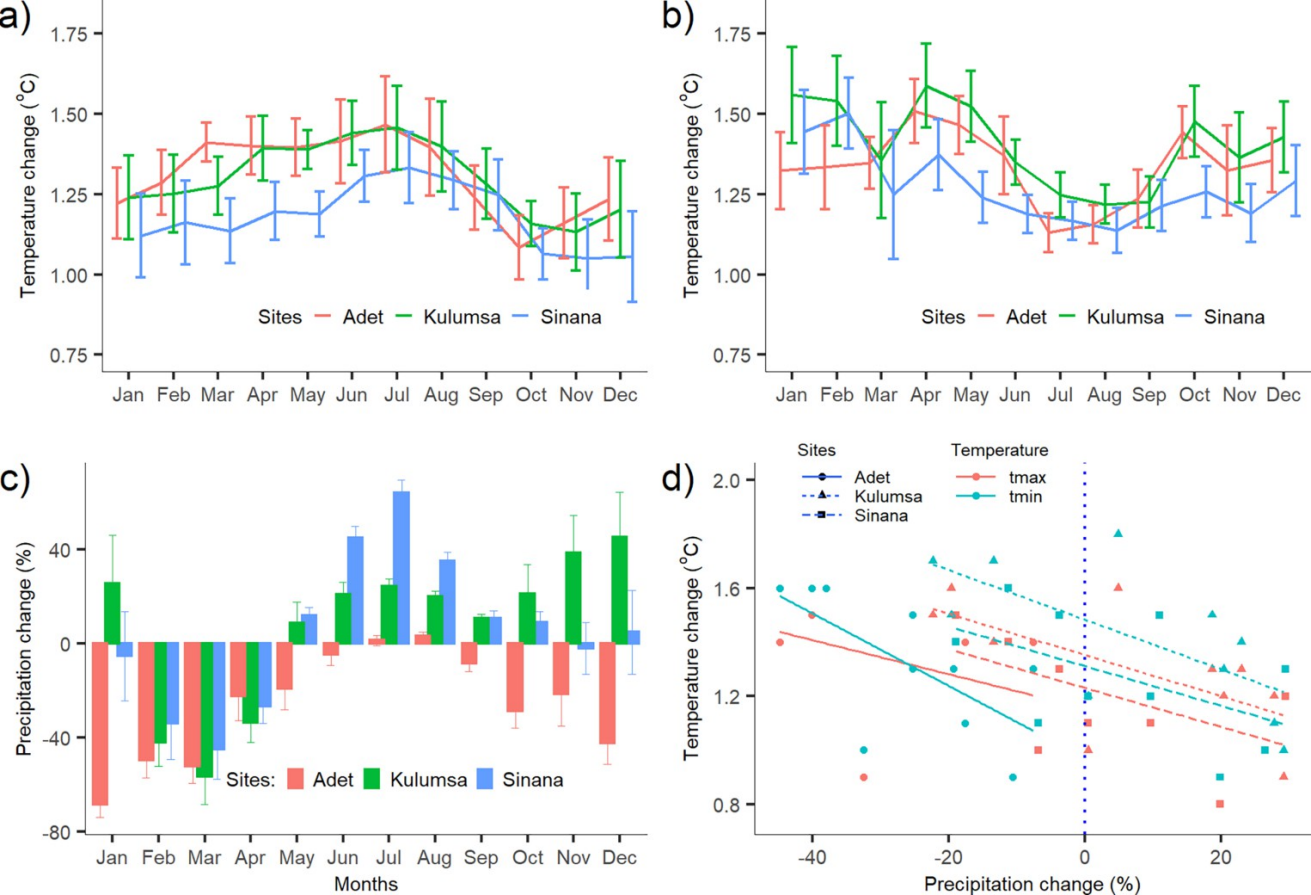
Changes from 1981–2010 in monthly average (a) maximum temperature (°C), (b) minimum temperature (°C), and (c) relative change in monthly averaged total precipitation (%) predicted by 2050 according to the 10 downscaled GCMs as compared to the baseline. Standard deviations shown as whiskers. Scatter plot between projected mean annual changes in maximum and minimum temperature and precipitation (d).

Compared to the baseline climate (1981–2010), a consistent temperature increase is expected by 2050 for all study sites whereas the change in precipitation is expected to differ, both seasonally as well as between sites. Averaged over the climate models, both annual maximum and minimum temperature are projected to increase by about 1.2–1.4°C by 2050 where the projected change in the minimum temperature would be slightly higher than that of the maximum temperature (Fig 2). Averaged across climate models, a slight increase in total annual precipitation is projected by 2050 in Sinana (+6%) and Kulumsa (+7%), whereas a decrease (>25%) is projected in Adet. However, the projected changes are highly variable among the climate models as well as between seasons (Fig 2C). For the growing season only, an increase by 17% and 29% in Kulumsa and Sinana, respectively, and a -2% in Adet are projected. Generally, a negative relationship was evident between the projected changes in temperature and precipitation with a majority of GCMs predicted hot and dry condition (Fig 2D).

### Model description

#### The Expert-N modelling package

**Fig 3:**
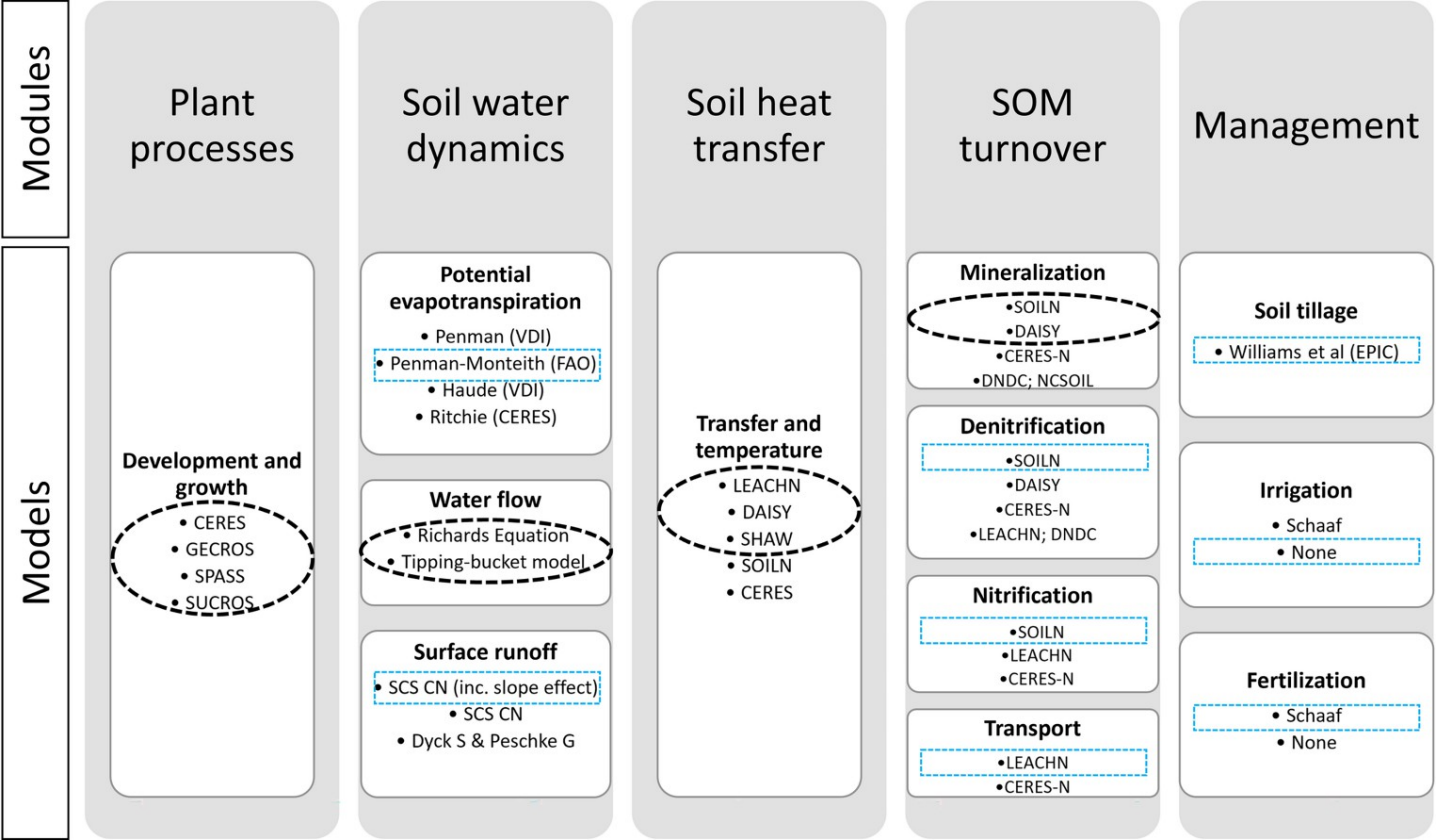
Schematic diagram of the modular components of the Expert-N agro-ecosystem modeling package. Submodels considered in the present study are surrounded by dashed lines. The circles show the submodels selected for factorial combination to form the multi-model whereas the boxes show the submodels that were constant to the multi-model.

The agro-ecosystem modelling package Expert-N provides process-based models and submodules for the description of coupled processes in the soil-plant system (Fig 3). This enables the simulation of saturated and unsaturated water flux, soil heat transfer, C and N turnover in the soil, evapotranspiration, transport of mineral nitrogen, and plant processes, given abiotic boundary conditions as well as soil and crop management [39]. Its modular design facilitates multiple model configurations by combining various submodels. The crop growth submodel enables the dynamic simulation of phenological development, crop growth, canopy photosynthesis, biomass accumulation, leaf area and root distribution, root water and nitrogen uptake, and yield.

#### Model ensembles

There exists a multitude of crop models and modelling approaches for simulating processes in agroecosystems. In many cases, there is no clear reason for preferring one process representation over another. It also depends on the data situation whether it is possible to decide a priori whether certain process formulations are to be preferred. In fact, many submodels were developed specifically for limited data availability. In our study, we followed a multi-model approach whenever it was not possible to decide a priori which approach would be superior given our data. In the present study, a multi-model ensemble (n = 48; Fig 3) was formed by coupling four crop growth submodels (CERES, SUCROS, SPASS, and GECROS), two soil organic matter turnover submodels (SOILN and DAISY), two soil water transport submodels (based on Richards equation and the tipping-bucket (TB) approach) and three soil heat transfer submodels (LEACHN, DAISY and SHAW).

The four selected crop growth submodels took part in the world-spanning agricultural model intercomparison and improvement project (AgMIP) for wheat [15,40] and maize [13,41] under contrasting conditions and environments across the globe. The models covered a good space of approaches and methods used in many common models. Most state-of-the-art crop models originate from two crop modelling schools, the DSSAT models and the Wageningen models. In this study, we used a further developed version of the CERES model based on an earlier version of DSSAT, two models from Wageningen (SUCROS and GECROS), and the hybrid model SPASS. Table 3 presents short summaries of the main features of the four crop growth models and their differences. The models vary in the level of detail and complexity of growth process representation [42]. The CERES was designed to simulate management and environmental effects on crop growth and development [43] and tested in different environments around the world [44]. SUCROS originally developed to simulate potential crop production with unlimited supply of plant nutrients [45], was combined in Expert-N with submodules for nitrogen uptake from WAVE [46] to take into account possible N-limitation effects on photosynthesis and crop growth. SPASS was developed by adopting approaches mostly from CERES and SUCROS with some new own functions [47]. GECROS is relatively a recent model. It is based on the SUCROS model and uses a set of new algorithms that enhanced the description of genotype-environment interaction [48]. Photosynthesis is calculated based on biochemical approach. This sets GECROS apart from the rest of the models that use less advanced approaches. Whereas SPASS was originally developed in Expert-N [49,50], the other crop growth models were implemented from freely available code or implemented based on their documentations. Therefore, they may slightly differ from their original version with regard to the coupling to soil modules of Expert-N (water uptake, nutrient uptake, litter transfer to soil organic matter pools). Short summaries of the main features of the four plant models and their differences are provided by previous studies [42,51,52].

**Table 3:** Main approaches adapted in the four crop growth models to describe various growth processes.

Model	Root distribution over depth	Soil hydraulic resistance dependence	Root hydraulic resistance	Radiation attenuation	Leaf internal CO_2_	Assimilate partitioning	Leaf area growth	Factors for leaf senescence
**CERES**	Dynamic, senescence included	Soil moisture content and root length	Included	Big-leaf model	–	Priority scheme	Exponential during juvenile phase, followed by a phase with constant s_la_	Age, drought, and nitrogen
**SUCROS**	Exponential decrease in four steps	Soil moisture content	–	Multilayer (three layers)	From constant $\frac{C_{i}}{C_{a}}$-ratio	Fixed allocation pattern	As in CERES	Age, self-shading, temperature
**SPASS**	As in CERES	As in CERES	Included	Multilayer (five layers)	As in SUCROS	Fixed allocation pattern	From leaf biomass growth rate, variable s_la_	Age, self-shading, temperature, and N
**GECROS**	Uniformly distributed	Not considered until permanent wilting point	–	Two-leaf model	Coupled to photosynthesis	Optimizing root and shoot activity	Depending on C or N limitation	N translocation from leaves to grains

Source [42].

The two soil organic matter turnover submodels included here were based on the SOILN model [53] and DAISY [54,55] model. Both use a multi-pool structure of soil organic matter. SOILN distinguishes three pools with distinct turnover rates (humus, litter, and manure) whereas DAISY considers six of them. In DAISY, the soil organic matter is divided into three main pools namely: added organic matter, soil microbial biomass and native soil organic matter where each pool is further divided into two parts, fast and slow. Turnover is determined by the availability of fractions for the soil organisms [56]. Descriptions of the implementations of SOILN and DAISY in Expert-N can be found in Berkenkamp et al. (2002).

The three soil heat transfer modules included were taken from LEACHN [57], DAISY [54] and SHAW [58]. These models differ in the terms considered in the general heat transfer equation, the numerical solution of the applied equation and in the way the upper and lower boundary conditions are defined. In LEACHN, the upper boundary condition is given by interpolation of average weakly air temperatures and average weakly temperature amplitudes. It is assumed that there is no heat flux across the lower boundary in 2 m soil depth. Convective heat flow with water and vapor is neglected in LEACHN; however, it is considered in the other models. In addition, SHAW considers the effects of soil frost and evaporation on the heat storage in soil. In both DAISY and SHAW, it is assumed that soil temperature at zero soil depth equals air temperature. At the bottom boundary, an analytical solution of the heat transfer equation is used assuming that frost/thaw and convective heat transfer can be neglected.

The two approaches used to simulate soil water dynamics were based on a numerical solution to the Richards equation, as implemented in the HYDRUS 1D model [59], and alternatively, on the tipping-bucket (TB) approach of CERES [60]. The former approach is based on the numerical solution of the governing water flow equation using an implicit finite element scheme, which can be used in unsaturated, partially saturated, or fully saturated porous media. It considers the gradients of soil water potential to simulate soil water movement. The tipping-bucket (TB) approach divides the soil into a cascade of ‘‘buckets” and calculates the water balance processes. Drainage occurs only when the field capacity of the overlying bucket is reached. Since the matric potential is neglected, upward soil water movement (capillary rise) is not simulated. Potential evapotranspiration was simulated based on the standard Penman-Monteith FAO56 method with a single crop coefficient [61] for all model ensemble members. The majority of the crop models participated in previous AgMIP studies were based on Penman-Monteith method [41,62]. Webber et al. [63] showed that the difference in methods to calculate the reference evapotranspiration (ET0) can result in significant uncertainty in yield estimates. However, the Penman-Monteith method is physically the most comprehensive method for calculating evapotranspiration. It consistently provides better estimates than other methods, in particular including the Penman equation [64] provided all input variables are available.

### Model calibration

The crop growth models were calibrated by minimizing the sum of squared errors between the model simulated and the observed experimental data. The data used for the calibration were flowering and maturity dates, and crop yields for six years for each cultivar (Table 1). The calibration procedure involved two steps. First, the genetic parameters (S2 Table) controlling simulated phenology were estimated based on observed flowering and maturity dates. In a second step, the parameters from step 1 were fixed, and the genetic parameters which control crop growth were estimated. For parameter estimation, we used the freely available inverse modelling code UCODE [65].

### Statistical analysis

#### Performance metrics

The performance of the calibration was evaluated using most commonly used statistical methods such as Pearson correlation coefficient (r), Root Mean Square Error (RMSE), the percent bias (PBIAS) and Nash-Sutcliffe efficiency (NSE). As a measure of agreement between simulated (sim) and observed (obs) data, we computed r (Eq 1). A value of r close to 1(-1) shows a perfect positive (negative) correlation between the simulated and observed data.


$$r=\frac{\sum_{i=1}^{N}\left({sim}_{i}-\bar{sim}).({obs}_{i}-\bar{obs}\right)}{\sqrt{\sum_{i=1}^{N}{\left({sim}_{i}-\bar{sim}\right)}^{2}}.\sqrt{\sum_{i=1}^{N}{\left({obs}_{i}-\bar{obs}\right)}^{2}}}$$ (1)

$$RMSE=\sqrt{\frac{\sum_{i=1}^{N}{\left({sim}_{i}-{obs}_{i}\right)}^{2}}{N}}\;x\;100$$ (2)

PBIAS (Eq 3) measures the extent to which the simulated values are larger or smaller compared to the observed values; positive (negative) values correspond to overestimation (underestimation) [66].


$$PBIAS=\frac{\sum_{i=1}^{N}\left({sim}_{i}-{obs}_{i}\right)}{\sum_{i=1}^{N}{obs}_{i}}\;x\;100$$ (3)

The NSE (Eq 4) measures the magnitude of the residual variance relative to the measured data variance [67]:

$$NSE=1-\frac{\sum_{i=1}^{N}{\left({sim}_{i}-{obs}_{i}\right)}^{2}}{\sum_{i=1}^{N}{\left({obs}_{i}-\bar{obs}\right)}^{2}}$$ (4)

where *obs* and *sim* refer to observed and simulated data whereas, $\bar{obs}$ and $\bar{sim}$ refer the average values respectively. NSE can range from −∞ to 1 where NSE = 1 corresponds to a perfect simulated data match, whereas NSE < 0 occurs when the mean of observed data describes the data better than the model simulation.

In addition, the calibration results were visualized in Taylor diagrams [68]. A Taylor diagram summarizes how well model simulation match the observed system behavior to facilitate the relative assessment of different models. For this, it combines three statistics in a 2-D graph: the normalized standard deviations (SD) of both simulated and observed data, Pearson’s correlation coefficient between simulated and observed data, and the centered RMSE. The radial distance (blue contours) from the point on the x-axis identified as "observation" is a measure of the centered RMSE between the simulated and observed. The normalized SD of the simulated pattern are proportional to the radial distance from the origin to the points and the cosine of the polar angle (pink) is equal to the correlation between simulated and observed data. The standardization of SD is done using the SD of the respective observational data so that the reference has a standard deviation of 1. The centered RMSE refers that the simulated and observed data are normalized by subtracting the respective means before calculating the RMSE.

#### Sensitivity analysis

To identify the most influential factors that affect grain yield, a sensitivity analysis was conducted by discretely varying selected input variables and analyzing their impact on modelled yield for the 30 baseline years (1981–2010). We considered five levels of fertilizer applications: 0, 40, 80, 120 and 160 kg ha^-1^; five [CO_2_] levels: 360, 450, 540, 630 and 720 ppm; four levels of temperature increase: 0, 2, 4, and 6°C; and five levels of precipitation change: -50, -25, 0, 25, and 50%. The perturbations are applied as absolute offsets for temperature treatments from daily maximum and minimum temperature data whereas precipitation perturbations are applied as fractional changes to daily data. The sensitivity to changes in [CO_2_], temperature and precipitation were evaluated under three (0, 80, 160 kg ha^-1^) N-fertilizer levels to evaluate their combined effects on grain yield. Here, the main purpose of the sensitivity analysis was to sample over a wide range of treatment space to understand the influential factors that drive grain yield changes. The levels were selected one to encompass the current situation (represented by 360 [CO_2_], 0°C temperature increase, and 0% precipitation increase from the baseline climate) and second to approximate as much as possible to the levels used in previous studies [13,41,69] for comparison purpose. Grain yield was simulated for 30 years * 141 perturbations * 48 model ensembles resulted in 67,680 simulations per cultivar. We analyzed the effect of the treatments using Student’s t-test and linear modelling with analysis of variance (ANOVA) and post-hoc Tukey test.

#### Climate change impact calculation

The potential impact of climate change on crop growth (yield and grain [N]) were computed by comparing the relative changes in the simulations driven by baseline (1981–2010) climate and future (2021–2050) climate projections. The relative percentage change (Δ*Z*) due to climate change was calculated as:

$$\Delta Z\;(\%)=\frac{{\bar{Z}}_{future}-{\bar{Z}}_{baseline}}{{\bar{Z}}_{baseline}}*100$$ (5)

where ${\bar{Z}}_{future}$ and ${\bar{Z}}_{baseline}$ represent the simulated median values for the future and baseline climates, respectively.

*Dissecting sources of uncertainty*. In predicting climate change impact on crop growth, uncertainties may arise for a variety of reasons. In this study, we compared the contributions to the uncertainty in the projected impact of climate change on yield from i) climate models, and ii) Expert-N models and submodules selected to represent soil and crop processes. As in Asseng et al. [17], the uncertainties were quantified by calculating the respective coefficient of variation (CV) for each crop cultivar. For instance, to calculate the CV due to climate models for a certain crop cultivar, first the standard deviation of yield changes over the ten climate models was calculated for each combination of crop and soil models:

$$\sigma_{i,j,k,l}^{\left(Climate\;model\right)}=\sqrt{VAR(\Delta Z|M_{i},N_{j},O_{k},P_{l})}\;for\;i=1,2,\ldots ,m;j=1,2,\ldots ,n;k=1,2,\ldots ,o\;and\;l=1,2,\ldots ,p$$ (6)

where *ΔZ* is simulated grain yield change, *ΔZ* | M_i_, N_j_, O_k_, *P*_l_ represent the simulated yield change by plant models *M*_*i*_, soil water models N_j_, SOM models O_k_ and soil heat models *P*_*l*_, and m, *n*, *o* and *p* are the total number of plant models, soil water models, SOM models and soil heat models, respectively. Second, the resulting m × n × o × p standard deviations, $\sigma_{i,j,k,l}^{\left(Climate\;model\right)}$ were normalized by division with the overall average of simulated yield change, $\bar{\Delta Z}$, and expressed as percentages:

$${CV(\%)}_{i,j,k,l}^{\left(Climate\;model\right)}=\frac{\sigma_{i,j,k,l}^{\left(Climate\;model\right)}}{\bar{\Delta Z}}*100$$ (7)

The same procedures were repeated to calculate the CVs resulting from the use of different plant models, soil water models, SOM models and soil heat models.

While the trials on the two maize cultivars were conducted at one site, the two wheat cultivars were grown at two different sites. Since the maize and wheat sites are not the same, we present our study per cultivar. Specifically, this means that the sensitivity analysis, the climate impact study, and the uncertainty decomposition analysis were conducted per cultivar.

*Crop management assumptions*. Throughout our analysis, the same crop cultivars and management were assumed to evolve in both baseline and future periods (S3 Table). The planting window was calculated by taking the mean +/- 2SD of the planting dates used for model calibration. Planting was triggered on the first occasion from the beginning of planting window with 20 mm or more within a 3-day period and no dry spell exceeding 10 days in the following 30 days [70]. The last day in the particular window will be set as a planting date if the condition was not met. In the present study, the models were run with same initial conditions each year. In addition, the pressure from pest and disease were not considered here. The effects of CO_2_ fertilization were not included in our climate change impact assessments.

## Results

### Model performance

The detail statistics (r, NSE, RMSE and PBIAS) of individual models’ performances are presented in S4 and S5 Tables. In the case of phenology (flowering and maturity dates), only the crop growth submodels affect the calibrations (S4 Table and S1 Fig). The RMSEs for the flowering dates were around two and five days for wheat and maize cultivars, respectively, and the corresponding correlation coefficient was higher than 0.90. The NSE values were also reasonably good ranging from 0.62 to 0.90 and from 0.6 to 0.94 for wheat and maize cultivars, respectively, where the PBIAS was below 3.2% in both crops. Similarly, close agreements were observed between the simulated and observed maturity dates. The models simulated maturity dates with RMSE lower than 2 and 5 days for wheat and maize cultivars, respectively, with r values greater than 0.97. The NSE values ranged from 0.53 to 0.96 and 0.8 to 0.98 for wheat and maize cultivars, respectively with a PBIAS value of below 2.0% in both crops. Overall, all simulated phenological stages (flowering and maturity dates) were in good agreement with the observations of both crops and in all cultivars.

**Fig 4:**
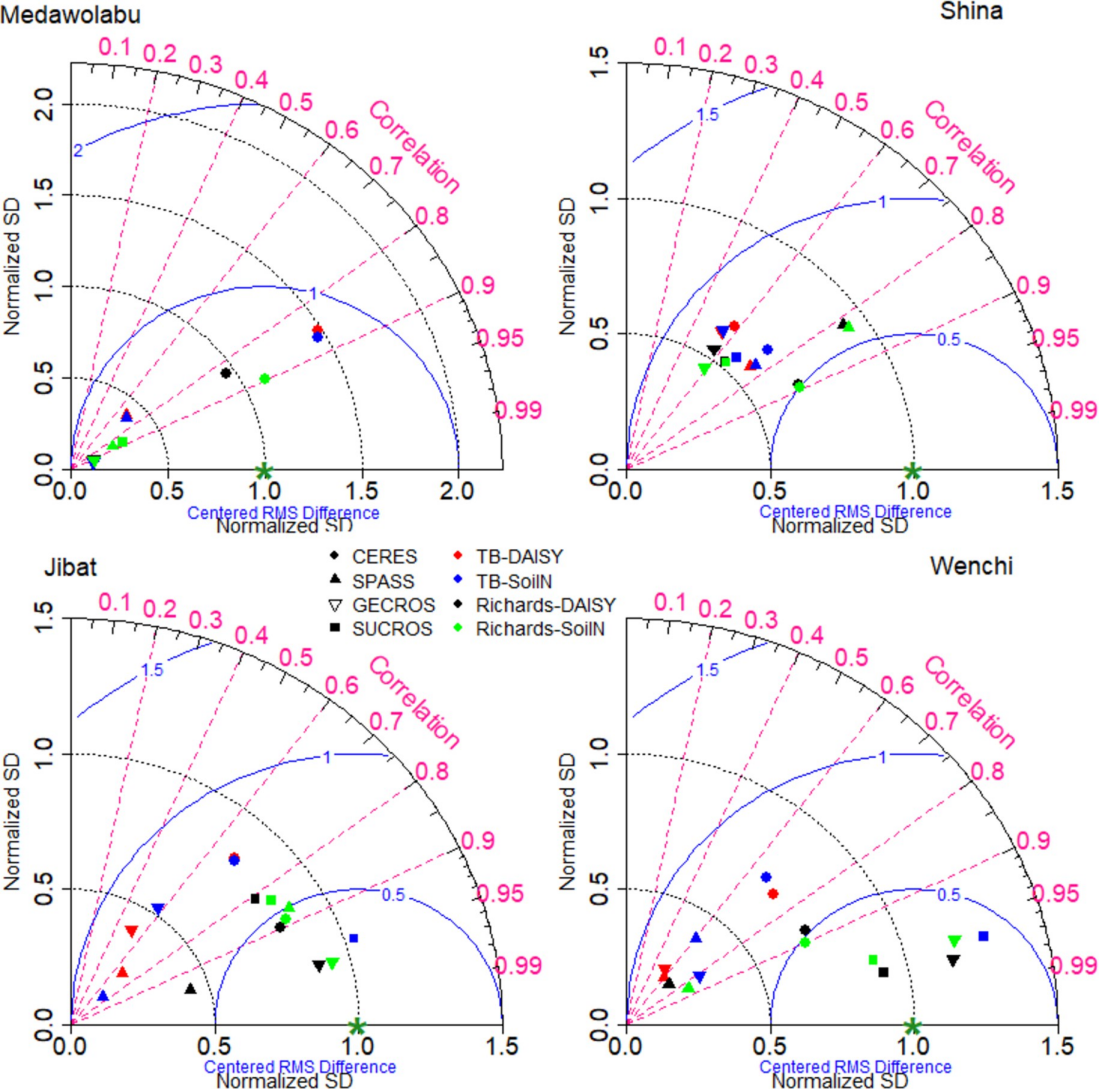
Taylor diagrams for simulated grain yield after calibration. Top and lower panel shows performance of model ensemble members for wheat and maize cultivars, respectively. The crop growth model estimates are represented by symbols: CERES (⚫), SPASS (▲), GECROS (▼) and SUCROS (■). Colors indicate tipping bucket combined with CN mineralization according to DAISY [red] and SOILN [blue] or Richards equation combined with CN turnover according to DAISY [black] and SOILN [green]), respectively. The green asterisks (*) marks the standard deviation of the observations (unity because of normalization). Centered RMSE values equal the distance between the simulated data points and the observation (green asterisk, *), readable from the blue lines.

The standard deviation, correlation coefficient, and RMSE (relative to the reference observation) for grain yield is shown Fig 4 and S5 Table. The variations due to soil heat transfer submodels were minimal and are not shown in the diagram. Each symbol and color combination represents a plant-soil water-soil organic matter model combination. For example, red circles represent simulation results obtained by running the CERES crop growth model together with the tipping-bucket approach for soil water movement and humus mineralization by DAISY. Other than with the phenological stages, the different ensemble model members showed quite large spread in simulating crop yields. The resulting ensemble members simulated grain yields of wheat and maize with RMSE values ranging from 0.13 t ha^-1^ to 0.54 t ha^-1^ and 0.36 t ha^-1^ to 1.01 t ha^-1^, respectively. The NSE values ranged from 0.21 to 0.75 and 0.15 to 0.95 for wheat and maize cultivars, respectively, where PBIAS values range approximately from -2% to +2% and -1.2% to +3.9%, respectively. The respective coefficient of correlation ranges from 0.51 to 0.90 and 0.4 to 0.97 for wheat and maize cultivars, respectively. Among the model ensemble, members that involved the GECROS plant model coupled with the tipping bucket approach perform less in simulating wheat grain yield. In contrast, model ensemble members based on the Richards equation reproduced the observed grain yield significantly better than those based on the tipping bucket approach. This holds for both crops.

### Sensitivity analysis

**Fig 5:**
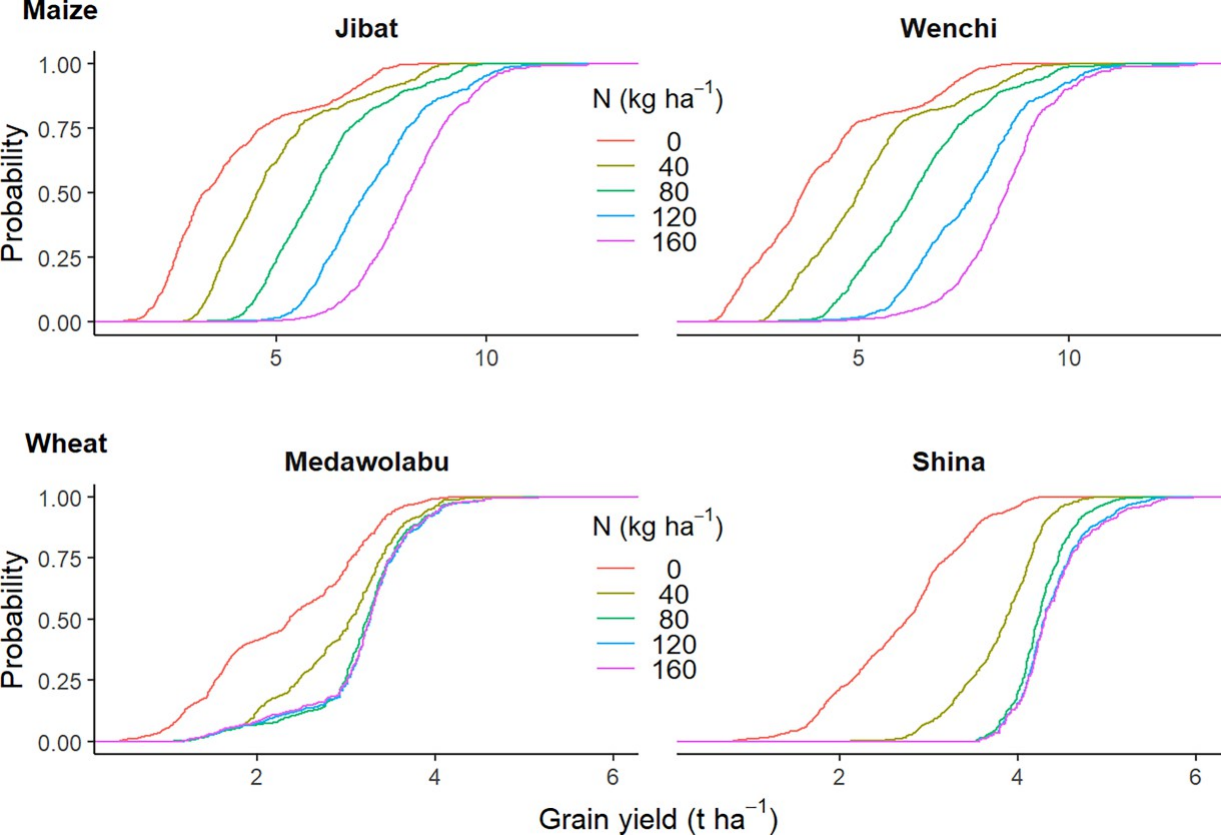
Probability of exceedance mean yield of ensemble models for the effects of different fertilizer levels.

The ‘probability-of–exceedance’ plots in Fig 5 show the spread of the ensemble runs for the simulated effect on grain yield, dependent on the different nitrogen fertilization application rates. Maize yield continuously increased with increasing fertilizer level, whereas the response of wheat grain yield was pronounced only until 80 kg ha^-1^N, after which the effect of additional fertilizer was small. Based on the ensemble median, increasing fertilizer application rates from 0 to 160 kg ha^-1^ could increase maize grain yield by 4 to 4.4 t ha^-1^ while the corresponding range of increase in wheat grain yield was 1.0 to 1.7 t ha^-1^. The variability in the ensemble was consistently higher in the lower fertilization levels across cultivars. For instance, at zero fertilization level, the standard deviation (SD) of the yields was more than 1.5 t ha^-1^ for maize and 0.8t ha^-1^ for wheat. However, the SD were less than 0.7 t ha^-1^ at the highest fertilization rate (160 kg ha^-1^) for both crops.

**Fig 6:**
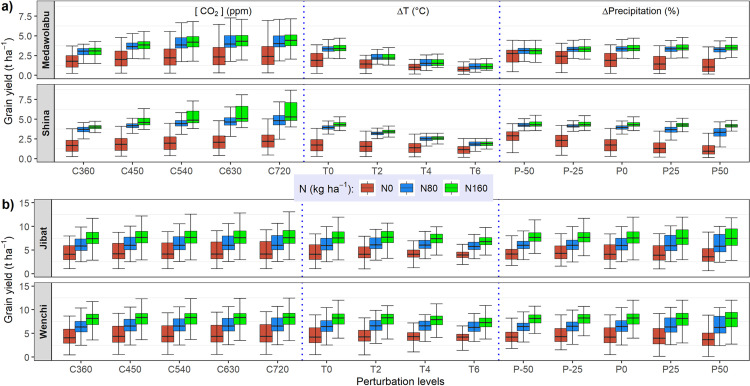
Effect of temperature (T), precipitation (P) and elevated CO_2_ (C) in air on grain yield for wheat (a) and maize (b) cultivars.

The results of the sensitivity analysis of grain yield to different levels of [CO_2_], temperature and precipitation are presented in box plots under three fertilizer levels (Fig 6). The associated multi-comparison ANOVA test result is also appended (S6 Table). The pattern of the effects of elevated CO_2_ were much clearer for wheat than for maize. The response of wheat grain yield to increased [CO_2_] were positive at all treatment levels and the effects were enhanced at higher fertilizer amounts. For instance, increasing [CO_2_] levels from 360 to 720 ppm increased median wheat grain yield by 1.2 t ha^-1^ under high fertilizer rates (160 kg ha^-1^) while the effect was only 0.5 t ha^-1^ for unfertilized treatment. However, the maize grain yield responded to a change from 360 to 450 ppm [CO_2_] level, beyond which no further response was observed. The corresponding maize grain yield increase was only 0.2 t ha^-1^ and the effects also show not much difference between different levels of fertilizer treatments.

Temperature had a clear effect on grain yield and the effect was stronger for wheat. Wheat grain yield decreased by 1.6 to 1.8 t ha^-1^ for a 6°C increase in temperature, which conforms with a relative decrease in yield of 47–57%. However, the effects on maize yield were modest. The decrease was less than 0.2 t ha^-1^ for a 6°C increase in temperature, which is less than 10%. In particular, there was no considerable difference in maize grain yield for 2°C temperature increase. The temperature effect on wheat yield was higher when N fertilization was high. A 6°C increase in temperature decreased the wheat yield by 50–60% under high N fertilization (160 kg ha^-1^) while without fertilizer, the corresponding decrease was 35–50%.

The simulated grain yields reacted rather insensitive to changes in precipitation. In some cases, a negative relationship between increases in precipitation and yield was simulated. A 50% decrease of precipitation resulted in a decrease of median maize yield by around 0.5 t ha^-1^ (p<0.05) while a 50% increase in precipitation led to no significant change. Wheat yield, on the other hand, show no significant response for precipitation change. However, despite its weak relative strength, wheat yield decreased by about 0.1 t ha^-1^ for a 50% decrease in precipitation under high N fertilization. There was also an instance where wheat yield decreased (increased) by 0.3 (0.2) t ha^-1^ for a 50% increase (decrease) in precipitation under unfertilized treatment. These results were opposite in tendency to those observed for the temperature and [CO_2_] treatments in the sense that the response decreased as N fertilization increased. Further investigation revealed that much of the effects of precipitation perturbation were attributed to N leaching which was relatively higher for wheat cultivars (S2 Fig). An increase in precipitation resulted in higher N leaching where the effects were different at different level of N input. The effects of increased precipitation on N leaching were larger at higher N fertilization rate (160 kg ha^-1^) for wheat cultivars while maize cultivars were more affected when N was limiting (0 kg ha^-1^). The results clearly show an interaction effect of nitrogen and precipitation. The pattern of precipitation effects was consistent between the maize cultivars but not between wheat cultivars.

### Impact of climate change on crop growth and development

**Fig 7:**
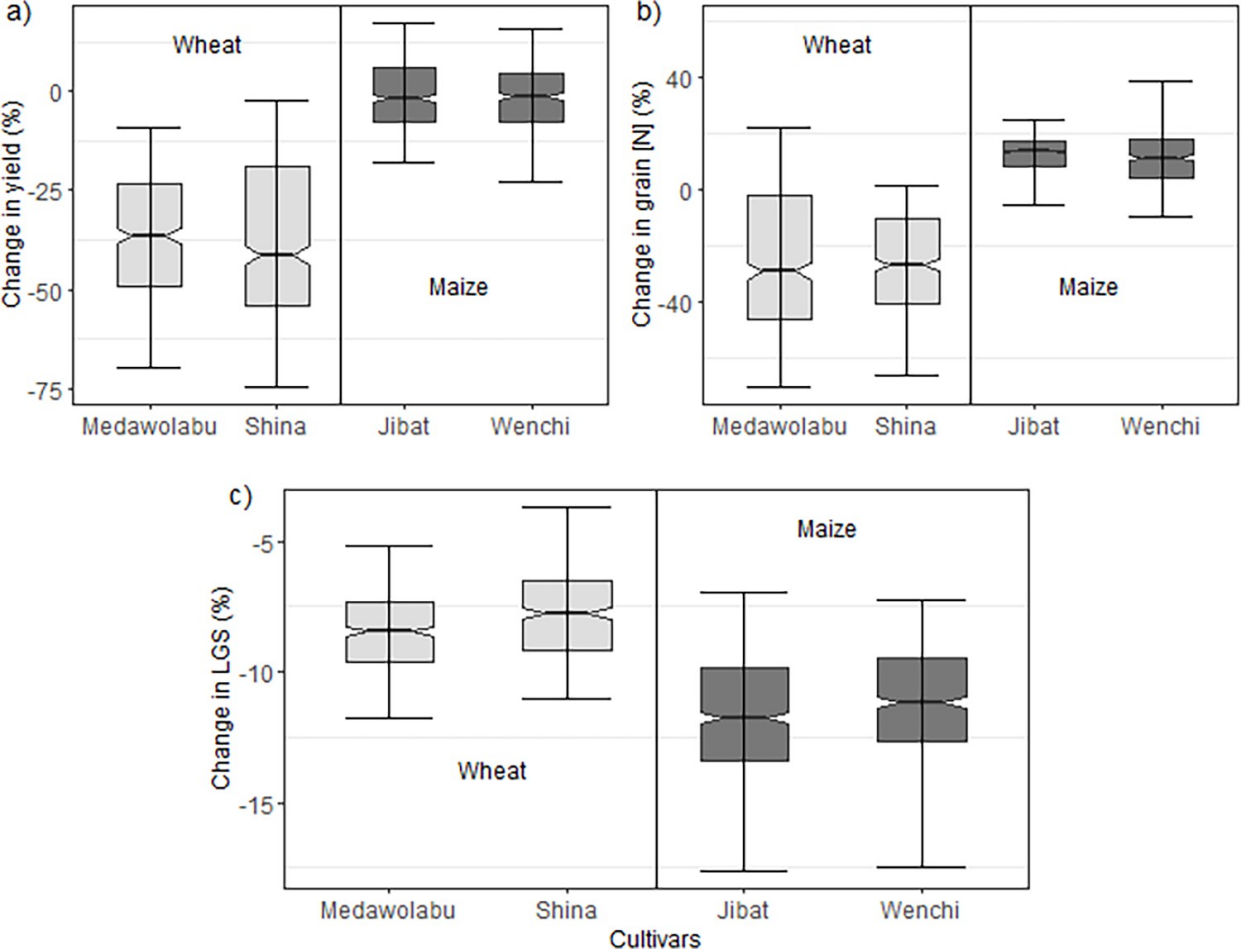
Relative change (%) in a) grain yield, b) grain N concentration and c) length of growing season (LGS) for the simulations of the multi-model ensemble for 2050s. The spread of the boxes shows the variations due to the multi-models.

Fig 7(A) presents the percentage change in grain yield between the baseline and future climate scenraios by the ensemble. Wheat grain yield is expected to decrease by 36 to 40% by 2050 whereas the impact on maize yield is minimal (~ -2%). However, the variability of the projected change in wheat grain yield was much higher SD ~ 18–21%. The results were consistent among the cultivars. The grain N concentration is expected to decrease by about 27% for wheat (Fig 7B). In contrast, the simulations suggest that maize grain N concentration will increase by 12 to 14%. The variability associated with the projected change of grain N was higher for wheat cultivars (SD: 17–28%) than for maize cultivars (SD: 7.5–15%). We analyzed the effect on the growing season length, as a definition for the operation window for the farmers. Due to climate change, a substantial shortening of crop life cycle (LGS) is projected for both crops (Fig 7C). A median shortening in LGS of 12 days was found for maize and between 8 to 9 days for wheat.

### Model uncertainties

**Fig 8:**
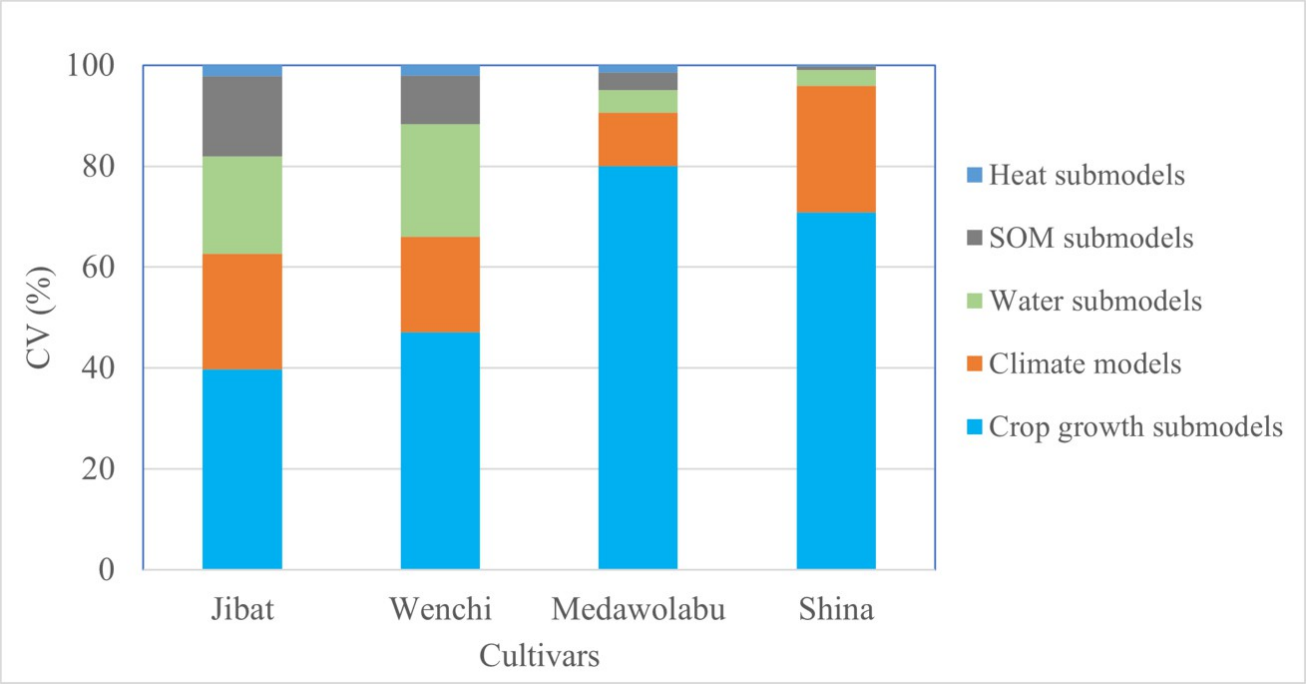
CVs (%) for the simulated grain yield changes according to the contribution by various model components for maize (right two bars) and wheat (left two bars) cultivars.

Fig 8 illustrates the contributions of different sources of uncertainty in the projected of grain yield change in the near future climate. The uncertainty in projected grain yield change caused by the variability in crop growth models was larger compared to the rest of the model components considered in this study. The corresponding median CVs were 40–80% (crop growth submodels), 11–25% (climate models), 3–22% (soil water flow submodels), 1–15% (soil organic matter submodels) and less than 2.5% (soil heat submodels). The uncertainty due to crop growth submodels was larger for wheat than maize. The uncertainty in the wheat grain yield changes was largely dominated by the variations in the crop growth models (with 71–80% CV) followed by climate models (with 11–25% CV). Meanwhile, the sources of uncertainty in the maize grain yield changes were fairly distributed among all model groups except for the soil heat submodels. Accordingly, 39–47% of the variations were attributed to the differences in crop growth submodels followed by climate models, soil water flow and soil organic matter submodels with CVs 19–23%, 19–22% and 10–16%, respectively. The contributions in the uncertainty were largely consistent within the crop cultivars but not across crop species. Among the model components considered, the uncertainty due to variations in the soil heat transfer submodels were minimal (CV<2.5%).

## Discussion

### Yield response to elevated CO_2_, warming atmosphere, change in precipitation and fertilizer application

It is established knowledge that elevated CO_2_ affects crop production. However, the strength of the response varies widely among crops and environment [71–76]. Compared to current yields, our study suggested that elevated CO_2_ to 720 ppm would increase wheat grain yield by 35–37% while maize grain yield would increase only by 3 to 5%, without considering the increase by the breeding progress or new technologies. Based on 59 peer-reviewed papers, Wang et al. (2013) conducted a meta-analysis on the impacts of elevated CO_2_ and found that wheat yield increased by 20–28% as [CO_2_] increased from 450 to 800 ppm. Depending on the environment, some studies even reported much higher yield increases of up to 44% [77] and 70% [73]. The effects of elevated CO_2_ were lower under low N fertilization treatments. The response of grain yield of C_3_ plants for elevated CO_2_ was higher under non-limiting N and H_2_O [74]. Regarding maize yield response, our results are consistent with previous modeling studies [13,78]. A doubling of [CO_2_] from 360 to 720 ppm resulted in an average 7.5% increase in maize grain yield [13]. The lower response of maize yield to increasing [CO_2_] in our study can be related to the site climate conditions, where rainfall is high. In C_4_ plants such as maize, the response of grain yield to increased levels of [CO_2_] is more pronounced under water stress conditions [72,75], due to water saving by a reduced stomatal conductance. Temperature, along with photosynthetic radiation and humidity are important agroclimatic factors that affect crop growth and development [79]. It affects various photosynthesis-related reactions and hence crop yield [80]. The present study highlights the negative effects of increasing temperature on grain yield. This is in-line with previous findings [81]. Higher temperature generally decreases yield by shortening the grain filling phase. Like the effects of elevated CO_2_, however, the level of the impact on wheat yield was much higher than that on maize yield. A temperature increase by 6°C from current climate reduces wheat grain yield by 47–57%, but the same temperature increment results in a reduction of only 10% in maize yield. The relative reduction in wheat yield was higher than that presented in Pirttioja et al. [82]. These authors reported 28% to 37% reduction in spring wheat yield over Europe in response to a 6°C temperature rise. However, in their global analysis, Asseng et al. [17] also found that wheat yield can be decreased by up to 56% in the tropics due to a 4°C increase. For maize, our results are comparable to the study of Bassu et al. [13]. Evaluating maize crop response to different climate factors using multi-crop models, they reported a modest reduction of grain yield.

The impact of precipitation on grain yield was the weakest in our sensitivity analysis. The effects of a 50% increase/decrease of current precipitation resulted in less than a 0.5 t ha^-1^ and 0.3 t ha^-1^ yield difference for maize and wheat. In some cases, precipitation increase had also a small negative effect on grain yield, particularly in case of wheat. These results have to be seen in the light that perturbation of precipitation did not affect the frequency of rainy or dry days but the quantity of rainfall. Guan et al. [83] also found similar decline in crop yield with increasing rainfall under high mean annual precipitation scenario in west Africa. Major cereal production including maize, wheat and sorghum showed no significant correlation with precipitation and should be studied together with other agronomic and climatic factors [84]. On the other hand, the effects of precipitation were smaller when considering increases in N fertilization which is in-line with the fact that application of N increases water use efficiency [85]. The simulated effects of precipitation changes were rather more pronounced in N leaching compared to grain yield. A possible explanation for the negligible effect of precipitation perturbation on grain yield could be attributed to the associated high soil organic carbon content at the study sites [86]. The high water holding capacity of the soils due to high organic carbon contents may provide the sufficient buffering capacity for extended time periods with low amounts of precipitation. More importantly, the low sensitivity could be attributed to the fact that the higher amount of precipitation at the study sites would be satisfactory even with -50% perturbations. Similar results were reported for sites in Africa (like Benin and Ethiopia) with high seasonal precipitation [41].

Results of the sensitivity analysis showed that nitrogen fertilization played a crucial role in modelled crop productivity in the study area. Compared to the unfertilized treatment, application of 160 kg ha^-1^ nitrogen increased maize and wheat median grain yield by 4.0–4.4 t ha^-1^ and 1.0–1.7 t ha^-1^, respectively, corresponding to a relative increase of 90 to 100% and 40 to 65%, respectively. In their field experiment conducted in a maize growing area in Ethiopia, Abera et al. [87] found up to 254% increase in maize grain yield with 110 kg ha^-1^ nitrogen application. For wheat, studies highlight that in the highlands of Ethiopia nitrogen application has even higher impact on grain yield [88].

### Possible effects of climate change on crop growth

Climate change will negatively affect crop production in Africa [6,89,90]. Our study suggests that future wheat yield will be a major challenge in Ethiopia (Fig 7A). However, the associated variability in the prediction of the grain yield was high. Averaged over all climate simulations, annual temperature is projected to increase by 1.2 to 1.4°C by 2050 in the study area (Fig 2A and 2B). This entails a tremendous impact on a cool season crop like wheat through the acceleration of phenological development [62]. Compared to the baseline period, the median length of the wheat life cycle is expected to shorten by eight to nine days by 2050 (Fig 7C). The exposure to climate extremes particularly during the grain filling phase [79] could probably explain the large yield decrease despite modest change in growing season duration which clearly worth further research. In their review of climate change impacts on major crops in eastern Africa, Adhikari et al. [91] highlighted wheat as the crop most vulnerable to climate change in the region. However, the projected yield losses could partly be offset if we included CO_2_ fertilization in our simulations, which have been found to have positive effects in C3 crops [76].

Studies suggest that atmospheric CO_2_ exposure also affects the availability of N and N use efficiency [80]. Our study indicate that the impact of changing climate also manifested itself in terms of grain quality where a decrease of up to 28% in wheat grain N concentration is projected by 2050 (Fig 7B) but with a high associated variability. Asseng et al. [15] evaluated the effect of climate change on wheat protein around the world using 32 different wheat crop models with combination of 5 GCMs. They found that grain protein concentration would decline as a result of climate change in most parts of Africa. The study suggested that reductions in grain quality were attributed to nitrogen availability limiting growth stimulus from elevated CO_2_ particularly in low-rainfall regions [15]. A meta-analysis study supported the result that elevated CO_2_ reduces grain quality albeit it stimulates wheat grain yield [92].

In contrast, our estimates showed maize production would not suffer as much as wheat crop with a projected grain yield reduction of less than 2% by 2050. This was even though maize growing season was projected to decrease by up to two weeks under future climate (Fig 7C). It suggests that changes in the critical development stages like the timing of flowering and length of grain filling period could be more important than the total growing season in determining the final grain yield. The projected increase in seasonal total precipitation for the site (Fig 2C) can also partly explain the minimal impact. The estimated relative change in maize grain yield is in line with the study conducted by Tesfaye et al. [10] who reported a relatively small yield reduction of 3–9% projected for 2050s for the highlands of Eastern Africa. Likewise, in their recent working paper, Timothy et al. [29] pointed out that the impact of climate change on crop yield including maize will be minimal. Some studies even reported that climate change would increase maize yield by 2050s. Araya et al. [30] studied the effects of climate change on maize yield in southwestern Ethiopia and found that median grain yield would increase by up to 4.2%. Generally, the projected impact of climate change on maize production would be minimum in east Africa [10,91,93]. In the lowland part of the country, however, much higher maize yield reduction (up to 20%) was projected by 2050 [32]. In contrast to the grain yield, climate change affected maize grain quality positively, where grain N concentration increased with median projection ranging from about 12% to 14% (Fig 7B).

### Sources of uncertainty

Understanding and quantifying different sources of uncertainties has become the core interest among scholars in multi-model climate change impact studies [13,20,62,78,94]. In this regard, sources including the choice of crop models and climate model scenarios contributed to the uncertainty of the simulated results. Comparisons of uncertainties among the different model components showed that differences in crop growth models resulted in largest uncertainties in the crop yield change estimates (Fig 8). Our result is in line with similar previous studies [17,95–97]. In the largest global crop model intercomparison exercise, Asseng et al. [17] compared 30 different crop models in simulating wheat growth around the world under rising temperature. They found that the uncertainty in simulated grain yield due to differences in crop models were more than the uncertainty caused by the variations in locations as well as the inter-annual variability. Similarly, in their climate change impact assessment, Araya et al. [95] reported the choice of crop models to cause much of the variations in projected maize yield.

The present study also shows that the uncertainties vary among crop species and cultivars as well (Fig 8). The uncertainties in the projected yield change due to crop growth models were higher for wheat than maize. This is related to the larger sensitivity of the wheat plant models and the magnitude of impact of climate change on wheat. The uncertainty in simulated wheat yield increased with increasing temperature [17]. The relative inconsistent pattern among the wheat cultivars also suggested that the contributions of different sources of uncertainties might also be site dependent [98]. The variation due to soil water dynamics submodules could be related with the different performance of the two approaches suggesting the importance of soil processes in such studies [99]. Our study showed that soil water dynamic submodule based on Richards equation approach performed better than the tipping bucket based approach in simulating yield. The result suggest that Richards based soil dynamics modeling approach would help in capturing the high variability in the observation data like in the case of ours. Richards equation based approach has a potential for better simulation of soil water dynamics provided that the necessary data are available [100]. Our study highlighted that a considerable amount of uncertainty originated from the different soil water flow and soil organic matter models. However, the variance in projected yield changes due to variations in crop growth models remain the main source of uncertainty across examined crop species and cultivars. This is mainly due to the fact that crop growth models vary largely in their approach to represent key dynamic processes [42] and overall, their predictive skill might still be limited. Model improvement has been hindered by unavailability of high-quality data as well as uncomplete understanding of some plant processes [101].

## Conclusions

In the face of climate change, agricultural decision making is and will be a challenging task due to the uncertainties associated with impact assessments. Different sources of uncertainties, including choice of crop models and climate model projections, challenge results of climate change impact assessment. Our results indicate that the use of multiple dynamic crop models provides a better understanding of the uncertainties as a consequence of model structural differences, leading to more robust analyses. The current study also included the uncertainties that could arise from future climate prediction through involving a variety of distinct climate models that were downscaled to finer resolution. In such an effort to quantify, dissect and compare uncertainties, the Expert-N agro-ecosystem modeling package was an ideal framework to facilitate multiple model configurations. Importantly, our results identified crop growth model associated uncertainty to be larger than the rest of model components considered in this study. Uncertainties varied between the crop species and cultivars as well. Incorporating different soil water flow and soil organic matter modeling approaches in multi-model ensemble uncertainty studies could also reveal a considerable portion of uncertainty in grain yield changes. Under the current crop management scenario, our results suggest that maintaining current wheat yield will be a challenging task for the region due to the projected greater impacts by 2050. To abate the associated problems, proper management strategies will be required. In this regard, proper application of N fertilization would alleviate part of the projected impact. Regarding precipitation, our sensitivity study was based on simple perturbations of the quantities to which the crops did not show a significant sensitivity. Possibly, the distribution of rainy days within the year might matter more than the difference in rain amounts in the region. In summary, our study quantifies the impact of climate change and demonstrates the importance of multi-model ensemble approach in understanding the associated uncertainties.

## Supporting information

S1 Fig Scatter plot between model simulation and observation for days to flowering and days to maturity for the four plant growth submodels for maize (a) and wheat (b) cultivars.
(TIF)

S2 Fig Effects of precipitation perturbations relative to the baseline climate on cumulative N leaching for (a) wheat and (b) maize cultivars.
(TIF)

S1 Table List of global climate models used for the climate change predictions. (DOCX)

S2 Table List of plant development and growth submodel parameters used for model optimization. (DOCX)

S3 Table Crop management data applied at the experimental sites. (DOCX)

S4 Table Summary of the different performance metrics for model calibration for phenology. It lists the performance of the four of plant growth submodels for each crop cultivar.
(DOCX)

S5 Table Summary of the different performance metrics for model calibration for yield. It lists the performance of the factorial combination of plant growth x soil water flow x soil organic matter submodels and modules.
(DOCX)

S6 Table ANOVA table for the response of grain yield to N fertilizer, CO2, temperature, and precipitation treatments. (DOCX)

S1 Text (DOCX)

S1 Dataset (CSV)

S2 Dataset (CSV)

S3 Dataset (XLSX)

S4 Dataset (CSV)

S5 Dataset (XLSX)

S6 Dataset (CSV)

S7 Dataset (XLSX)

## References

[1] Howden SM, Soussana J-F, Tubiello FN, Chhetri N, Dunlop M, Meinke H. Adapting agriculture to climate change. Proc Natl Acad Sci U S A. 2007;104: 19691–19696. doi: 10.1073/pnas.0701890104 18077402 PMC2148359

[2] Xiong W, Reynolds MP, Crossa J, Schulthess U, Sonder K, Montes C, et al. Increased ranking change in wheat breeding under climate change. Nat Plants. 2021;7: 1207–1212. doi: 10.1038/s41477-021-00988-w 34462575

[3] Thornton PK, Ericksen PJ, Herrero M, Challinor AJ. Climate variability and vulnerability to climate change: A review. Glob Chang Biol. 2014;20: 3313–3328. doi: 10.1111/gcb.12581 24668802 PMC4258067

[4] IPCC. Summary for Policymakers. Global Warming of 1.5°C. An IPCC Special Report on the impacts of global warming of 1.5 oC above pre-industrial levels and related global greenhouse gas emission pathways,. Context Strength Glob response to Threat Clim Chang Sustain Dev efforts to eradicate poverty. World Meteorological Organization, Geneva, Switzerland; 2018. Available: https://www.ipcc.ch/site/assets/uploads/sites/2/2018/07/SR15_SPM_version_stand_alone_LR.pdf.

[5] Gammans M, Mérel P, Ortiz-Bobea A. Negative impacts of climate change on cereal yields: Statistical evidence from France. Environ Res Lett. 2017;12: 54007. doi: 10.1088/1748-9326/aa6b0c

[6] Knox J, Hess T, Daccache A, Wheeler T. Climate change impacts on crop productivity in Africa and South Asia. Environ Res Lett. 2012;7: 34032. doi: 10.1088/1748-9326/7/3/034032

[7] Ruppel Niang I, O.C., Abdrabo M.A., Essel A., Lennard C., Padgham J., et al. Africa. In: Barros VR, Field CB, Dokken DJ, Mastrandrea MD, Mach KJ, Bilir TE, et al., editors. Climate Change 2014. Cambridge, United Kingdom and New York, NY, USA: Cambridge University Press; 2014. pp. 1199–1265.

[8] Van Ittersum MK, Van Bussel LGJ, Wolf J, Grassini P, Van Wart J, Guilpart N, et al. Can sub-Saharan Africa feed itself? Proc Natl Acad Sci U S A. 2016;113: 14964–14969. doi: 10.1073/pnas.1610359113 27956604 PMC5206509

[9] FAO and ECA. Regional Overview of Food Security and Nutrition: Addressing the threat from climate variability and extremes for food security and nutrition. Acra, Ghana; 2018.

[10] Tesfaye K, Gbegbelegbe S, Cairns JE, Shiferaw B, Prasanna BM, Sonder K, et al. Maize systems under climate change in sub-Saharan Africa. Int J Clim Chang Strateg Manag. 2015;7: 247–271. doi: 10.1108/IJCCSM-01-2014-0005

[11] Schlenker W, Lobell DB. Robust negative impacts of climate change on African agriculture. Environ Res Lett. 2010;5: 14010. doi: 10.1088/1748-9326/5/1/014010

[12] Chen S, Chen X, Xu J. Impacts of climate change on agriculture: Evidence from China. J Environ Econ Manage. 2016;76: 105–124. doi: 10.1016/j.jeem.2015.01.005

[13] Bassu S, Brisson N, Durand J-L, Boote K, Lizaso J, Jones JW, et al. How do various maize crop models vary in their responses to climate change factors? Glob Chang Biol. 2014;20: 2301–2320. doi: 10.1111/gcb.12520 PM– . 24395589

[14] Tao F, Rötter RP, Palosuo T, Gregorio Hernández Díaz-Ambrona C, Mínguez MI, Semenov MA, et al. Contribution of crop model structure, parameters and climate projections to uncertainty in climate change impact assessments. Glob Chang Biol. 2018;24: 1291–1307. doi: 10.1111/gcb.14019 29245185

[15] Asseng S, Martre P, Maiorano A, Rötter RP, O’Leary GJ, Fitzgerald GJ, et al. Climate change impact and adaptation for wheat protein. Glob Chang Biol. 2019;25: 155–173. doi: 10.1111/gcb.14481 PM– . 30549200

[16] Holzkämper A, Calanca P, Fuhrer J. Identifying climatic limitations to grain maize yield potentials using a suitability evaluation approach. Agric For Meteorol. 2013;168: 149–159. doi: 10.1016/j.agrformet.2012.09.004

[17] Asseng S, Ewert F, Martre P, Rötter RP, Lobell DB, Cammarano D, et al. Rising temperatures reduce global wheat production. Nat Clim Chang. 2015;5: 143–147. doi: 10.1038/nclimate2470

[18] Challinor AJ, Smith MS, Thornton P. Use of agro-climate ensembles for quantifying uncertainty and informing adaptation. Agric For Meteorol. 2013;170: 2–7. doi: 10.1016/j.agrformet.2012.09.007

[19] Martre P, Wallach D, Asseng S, Ewert F, Jones JW, Rötter RP, et al. Multimodel ensembles of wheat growth: Many models are better than one. Glob Chang Biol. 2015;21: 911–925. doi: 10.1111/gcb.12768 25330243

[20] Xiong W, Asseng S, Hoogenboom G, Hernandez-ochoa I. Different uncertainty distribution between high and low latitudes in modelling warming impacts on wheat. Nat Food. 2019; 0–1. doi: 10.1038/s43016-019-0004-2

[21] Kimball BA, Boote KJ, Hatfield JL, Ahuja LR, Stockle C, Archontoulis S, et al. Simulation of maize evapotranspiration: An inter-comparison among 29 maize models. Agric For Meteorol. 2019;271: 264–284. doi: 10.1016/j.agrformet.2019.02.037

[22] Vereecken H, Schnepf A, Hopmans JW, Javaux M, Or D, Roose T, et al. Modeling Soil Processes: Review, Key Challenges, and New Perspectives. Vadose Zo J. 2016;15: vzj2015.09.0131. doi: 10.2136/vzj2015.09.0131

[23] Asseng S, Ewert F, Rosenzweig C, Jones JW, Hatfield JL, Ruane AC, et al. Uncertainty in simulating wheat yields under climate change. Nat Clim Chang. 2013;3: 827 EP-. doi: 10.1038/nclimate1916

[24] CSA. Agricultural Sample Survey 2017/2018. Volume I Report on Area and Production of Major Crops (Private Peasant Holdings, Meher Season). Addis Ababa, Ethiopia: Statistical Bulletin; 2018.

[25] Alemayehu A, Bewket W. Local climate variability and crop production in the central highlands of Ethiopia. Environ Dev. 2016;19: 36–48. doi: 10.1016/j.envdev.2016.06.002

[26] Esayas B, Simane B, Teferi E, Ongoma V, Tefera N. Trends in Extreme Climate Events over Three Agroecological Zones of Southern Ethiopia. Adv Meteorol. 2018;2018: 1–17. doi: 10.1155/2018/7354157

[27] Gebrechorkos SH, Hülsmann S, Bernhofer C. Changes in temperature and precipitation extremes in Ethiopia, Kenya, and Tanzania. Int J Climatol. 2019;39: 18–30. doi: 10.1002/joc.5777

[28] Gummadi S, Rao KPC, Seid J, Legesse G, Kadiyala MDM, Takele R, et al. Spatio-temporal variability and trends of precipitation and extreme rainfall events in Ethiopia in 1980–2010. Theor Appl Climatol. 2018;134: 1315–1328. doi: 10.1007/s00704-017-2340-1

[29] Timothy T, Paul D, Richard R. Climate Change Impacts on Crop Yields in Ethiopia. Washington, DC and Addis Ababa, Ethiopia; 2019. Available: 10.2499/p15738coll2.133104.

[30] Araya A, Kisekka I, Girma A, Hadgu KM, Tegebu FN, Kassa AH, et al. The challenges and opportunities for wheat production under future climate in Northern Ethiopia. J Agric Sci. 2017;155: 379–393. doi: 10.1017/S0021859616000460

[31] Abera K, Crespo O, Seid J, Mequanent F. Simulating the impact of climate change on maize production in Ethiopia, East Africa. Environ Syst Res. 2018;7. doi: 10.1186/s40068-018-0107-z

[32] Kassie BT, Asseng S, Rotter RP, Hengsdijk H, Ruane AC, van Ittersum MK. Exploring climate change impacts and adaptation options for maize production in the Central Rift Valley of Ethiopia using different climate change scenarios and crop models. Clim Change. 2015;129: 145–158. doi: 10.1007/s10584-014-1322-x

[33] Muluneh A, Biazin B, Stroosnijder L, Bewket W, Keesstra S. Impact of predicted changes in rainfall and atmospheric carbon dioxide on maize and wheat yields in the Central Rift Valley of Ethiopia. Reg Environ Chang. 2015;15: 1105–1119. doi: 10.1007/s10113-014-0685-x

[34] Ruane AC, Goldberg R, Chryssanthacopoulos J. Climate forcing datasets for agricultural modeling: Merged products for gap-filling and historical climate series estimation. Agric For Meteorol. 2015;200: 233–248. doi: 10.1016/j.agrformet.2014.09.016

[35] Giorgi F, Jones C, Asrar G. Addressing climate information needs at the regional level: the CORDEX framework. … Organization (WMO) Bulletin. Geneva: World Meteorological Organization (WMO); 2009. pp. 175–183. Available: http://www.euro-cordex.net/uploads/media/Download_01.pdf.

[36] Moss RH, Edmonds JA, Hibbard KA, Manning MR, Rose SK, van Vuuren DP, et al. The next generation of scenarios for climate change research and assessment. Nature. 2010;463: 747–756. doi: 10.1038/nature08823 20148028

[37] Räisänen J, Räty O. Projections of daily mean temperature variability in the future: Cross-validation tests with ENSEMBLES regional climate simulations. Clim Dyn. 2013;41: 1553–1568. doi: 10.1007/s00382-012-1515-9

[38] Willems P, Vrac M. Statistical precipitation downscaling for small-scale hydrological impact investigations of climate change. J Hydrol. 2011;402: 193–205. doi: 10.1016/j.jhydrol.2011.02.030

[39] Priesack E. Expert-N Dokumentation der Modellbibliothek–FAM-Bericht 60. Hieronymus, München, Germany; 2006.

[40] Wallach D, Palosuo T, Thorburn P, Hochman Z, Andrianasolo F, Asseng S, et al. Multi-model evaluation of phenology prediction for wheat in Australia. Agric For Meteorol. 2021;298–299: 108289. 10.1016/j.agrformet.2020.108289.

[41] Falconnier GN, Corbeels M, Boote KJ, Affholder F, Adam M, MacCarthy DS, et al. Modelling climate change impacts on maize yields under low nitrogen input conditions in sub-Saharan Africa. Glob Chang Biol. 2020;26: 5942–5964. doi: 10.1111/gcb.15261 32628332

[42] Wöhling T, Gayler S, Priesack E, Ingwersen J, Wizemann H-D, Högy P, et al. Multiresponse, multiobjective calibration as a diagnostic tool to compare accuracy and structural limitations of five coupled soil-plant models and CLM3.5. Water Resour Res. 2013;49: 8200–8221. doi: 10.1002/2013WR014536

[43] Ritchie JT, Godwin D. CERES Wheat 2.0. 1987. Available: https://nowlin.psm.msu.edu/wheat_book/.

[44] Basso B, Liu L, Ritchie JT. A Comprehensive Review of the CERES-Wheat, -Maize and -Rice Models’ Performances. Advances in Agronomy. Elsevier Inc.; 2016. doi: 10.1016/bs.agron.2015.11.004

[45] Van Laar HH, Goudriaan J, Van Keulen H. SUCROS97: Simulation of crop growth for potential and water-limited production situations. As applied to spring wheat. 122, Instituut voor Agrobiologisch en Bodemvruchtbaarheidsonderzoek,: AB-DLO, TPE; 1997. Available: https://edepot.wur.nl/4426.

[46] Vanclooster M, Viaena P, Christiaens K. WAVE, A mathematical model for simulating water and agrochemicals in the soil and vadose environment. Reference and user’s manual, release 2. K.U.Leuven, Belgium: Institute for Land and Water Management; 1994.

[47] Wang E, Engel T. SPASS: a generic process-oriented crop model with versatile windows interfaces. Environ Model Softw. 2000;15: 179–188.

[48] Yin X, van Laar HH. Crop systems dynamics: an ecophysiological simulation model for genotype-by-environment interactions. Wageningen, The Netherlands: Wageningen Academic Publishers; 2005.

[49] Gayler S, Wang E, Priesack E, Schaaf T, -X maidle F. Modeling biomass growth, N-uptake and phenological development of potato crop. Geoderma. 2002;105: 367–383.

[50] Wang E. Development of a Generic Process-oriented Model for Simulation of Crop Growth. Herbert Utz Verlag. 1997. Available: https://books.google.com.au/books/about/Development_of_a_Generic_Process_oriente.html?id=AkwpWWUqjtUC&redir_esc=y.

[51] Biernath C, Gayler S, Bittner S, Klein C, Högy P, Fangmeier A, et al. Evaluating the ability of four crop models to predict different environmental impacts on spring wheat grown in open-top chambers. Eur J Agron. 2011;35: 71–82. doi: 10.1016/j.eja.2011.04.001

[52] Priesack E, Gayler S, Hartmann HP. The impact of crop growth sub-model choice on simulated water and nitrogen balances. Nutr Cycl Agroecosystems. 2006;75: 1–13. doi: 10.1007/s10705-006-9006-1

[53] Johnsson H, Bergstrom L, Jansson P-E, Paustian K. Simulated nitrogen dynamics and losses in a layered agricultural soil. Agric Ecosyst Environ. 1987;18: 333–356. doi: 10.1016/0167-8809(87)90099-5

[54] Hansen S, Jensen HE, Nielsen NE, Svendsen H. DAISY—Soil Plant Atmosphere System Model. NPO Report No. A 10. The National Agency for Environmental Protection. 1990. pp. 1–276.

[55] Hansen S, Jensen HE, Nielsen NE, Svendsen H. Simulation of nitrogen dynamics and biomass production in winter wheat using the Danish simulation model DAISY. Fertil Res. 1991;27: 245–259. doi: 10.1007/BF01051131

[56] Abrahamsen P, Hansen S. Daisy: an open soil-crop-atmosphere system model. Environ Model Softw. 2000;15: 313–330.

[57] Hutson JL, Wagenet RJ. LEACHM: Version 3: A process-based model for water and solute movement, transformation, plant uptake and chemical reactions in the unsaturated zone. 1992.

[58] Flerchinger GN. The Simultaneous Heat and Water (SHAW) Model: Technical Documentation, Tech. Rep. NWRC–2000–09, USDA–ARS, Northwest Watershed Research Center, Boise, Idaho. 2000.

[59] Simunek J, Huang K, van Genuchten MT. The HYDRUS code for simulating the one-dimensional movement of water, heat, and multiple solutes in variably-saturated media. Version 6.0. 1998.

[60] Ritchie JT. A user-oriented model of the soil water balance in wheat. Day Wand Atkin R K (Ed)-Wheat growth and modelling Series A: Life Sciences. Plenum Press, NY SV—86; 1985.

[61] Allen RG, Pereira LS, Raes D, Smith M. Crop evapotranspiration—Guidelines for computing crop water requirements—FAO Irrigation and drainage paper 56. FAO, Rome, Italy; 1998. p. pp300.

[62] Asseng S, Foster I, Turner NC. The impact of temperature variability on wheat yields. Glob Chang Biol. 2011;17: 997–1012. doi: 10.1111/j.1365-2486.2010.02262.x

[63] Webber H, Gaiser T, Oomen R, Teixeira E, Zhao G, Wallach D, et al. Uncertainty in future irrigation water demand and risk of crop failure for maize in Europe. Environ Res Lett. 2016;11. doi: 10.1088/1748-9326/11/7/074007

[64] Muhammad MKI, Nashwan MS, Shahid S, bin Ismail T, Song YH, Chung ES. Evaluation of empirical reference evapotranspiration models using compromise programming: A case study of Peninsular Malaysia. Sustain. 2019;11. doi: 10.3390/su11164267

[65] Poeter EP, Mary CH, Dan L, Claire RT, Steffen M. UCODE_2014, with new capabilities to define parameters unique to predictions, calculate weights using simulated values, estimate parameters with SVD, evaluate uncertainty with MCMC, and more. Integrated Ground Water Modeling Center Report Number GWMI 2014–02 U6 - https://igwmc.mines.edu/ucode; 2014.

[66] Yapo PO, Gupta HV, Sorooshian S. Automatic calibration of conceptual rainfall-runoff models: Sensitivity to calibration data. J Hydrol. 1996. doi: 10.1016/0022-1694(95)02918-4

[67] Nash JE, Sutcliffe J V. River flow forecasting through conceptual models part I—A discussion of principles. J Hydrol. 1970;10: 282–290. doi: 10.1016/0022-1694(70)90255-6

[68] Taylor KE. Summarizing multiple aspects of model performance in a single diagram. J Geophys Res Atmos. 2001;106: 7183–7192. doi: 10.1029/2000JD900719

[69] Asseng S, Ewert F, Martre P, Rötter RP, Lobell DB, Cammarano D, et al. Rising temperatures reduce global wheat production. Nat Clim Chang. 2015;5: 143–147. doi: 10.1038/nclimate2470

[70] Stern RD, Cooper PJM. Assessing climate risk and climate change using rainfall data—A case study from Zambia. Exp Agric. 2011;47: 241–266. doi: 10.1017/S0014479711000081

[71] Ainsworth EA, Leakey ADB, Ort DR, Long SP. FACE-ing the facts: Inconsistencies and interdependence among field, chamber and modeling studies of elevated CO2 impacts on crop yield and food supply. New Phytol. 2008;179: 5–9. doi: 10.1111/j.1469-8137.2008.02500.x 18482226

[72] Durand J-L, Delusca K, Boote K, Lizaso J, Manderscheid R, Weigel HJ, et al. How accurately do maize crop models simulate the interactions of atmospheric CO2 concentration levels with limited water supply on water use and yield? Eur J Agron. 2018;100: 67–75. doi: 10.1016/j.eja.2017.01.002

[73] Fitzgerald GJ, Tausz M, O’Leary G, Mollah MR, Tausz-Posch S, Seneweera S, et al. Elevated atmospheric CO2 can dramatically increase wheat yields in semi-arid environments and buffer against heat waves. Glob Chang Biol. 2016;22: 2269–2284. doi: 10.1111/gcb.13263 PM– . 26929390

[74] Kimball BA. Crop responses to elevated CO2 and interactions with H2O, N, and temperature. Curr Opin Plant Biol. 2016;31: 36–43. doi: 10.1016/j.pbi.2016.03.006 PM– . 27043481

[75] Manderscheid R, Erbs M, Weigel H-J. Interactive effects of free-air CO2 enrichment and drought stress on maize growth. Eur J Agron. 2014;52: 11–21. doi: 10.1016/j.eja.2011.12.007

[76] Wang L, Feng Z, Schjoerring JK. Effects of elevated atmospheric CO2 on physiology and yield of wheat (Triticum aestivum L.): A meta-analytic test of current hypotheses. Agric Ecosyst Environ. 2013;178: 57–63. doi: 10.1016/j.agee.2013.06.013

[77] Manderscheid R, Weigel H-J. Drought stress effects on wheat are mitigated by atmospheric CO2 enrichment. Agron Sustain Dev. 2007;27: 79–87. doi: 10.1051/agro:2006035

[78] Makowski D, Asseng S, Ewert F, Bassu S, Durand JL, Li T, et al. A statistical analysis of three ensembles of crop model responses to temperature and CO 2 concentration. Agric For Meteorol. 2015;214–215: 483–493. doi: 10.1016/j.agrformet.2015.09.013

[79] Hatfield JL, Prueger JH. Temperature extremes: Effect on plant growth and development. Weather Clim Extrem. 2015;10: 4–10. doi: 10.1016/j.wace.2015.08.001

[80] Hussain S, Ulhassan Z, Brestic M, Zivcak M, Weijun Zhou, Allakhverdiev SI, et al. Photosynthesis research under climate change. Photosynth Res. 2021;150: 5–19. doi: 10.1007/s11120-021-00861-z 34235625

[81] Zhao C, Liu B, Piao S, Wang X, Lobell DB, Huang Y, et al. Temperature increase reduces global yields of major crops in four independent estimates. Proc Natl Acad Sci U S A. 2017;114: 9326–9331. doi: 10.1073/pnas.1701762114 28811375 PMC5584412

[82] Pirttioja N, Carter TR, Fronzek S, Bindi M, Hoffmann H, Palosuo T, et al. Temperature and precipitation effects on wheat yield across a European transect: A crop model ensemble analysis using impact response surfaces. Clim Res. 2015;65: 87–105. doi: 10.3354/cr01322

[83] Guan K, Sultan B, Biasutti M, Baron C, Lobell DB. What aspects of future rainfall changes matter for crop yields in West Africa? Geophys Res Lett. 2015;42: 8001–8010. doi: 10.1002/2015GL063877

[84] Admassu S. Rainfall Variation and its Effect on Crop Production in Ethiopia. Department of Civil Engineering, Addis Ababa University. 2004.

[85] Fan T, Stewart BA, Yong W, Junjie L, Guangye Z. Long-term fertilization effects on grain yield, water-use efficiency and soil fertility in the dryland of Loess Plateau in China. Agric Ecosyst Environ. 2005;106: 313–329. doi: 10.1016/j.agee.2004.09.003

[86] Wood SA, Tirfessa D, Baudron F. Soil organic matter underlies crop nutritional quality and productivity in smallholder agriculture. Agric Ecosyst Environ. 2018;266: 100–108. 10.1016/j.agee.2018.07.025.

[87] Abera T, Debele T, Wegary D. Effects of Varieties and Nitrogen Fertilizer on Yield and Yield Components of Maize on Farmers Field in Mid Altitude Areas of Western Ethiopia. Int J Agron. 2017;2017: 1–13. doi: 10.1155/2017/4253917

[88] Belete F, Dechassa N, Molla A, Tana T. Effect of nitrogen fertilizer rates on grain yield and nitrogen uptake and use efficiency of bread wheat (Triticum aestivum L.) varieties on the Vertisols of central highlands of Ethiopia. Agric Food Secur. 2018;7: 362. doi: 10.1186/s40066-018-0231-z

[89] Challinor A, Wheeler T, Garforth C, Craufurd P, Kassam A. Assessing the vulnerability of food crop systems in Africa to climate change. Clim Change. 2007;83: 381–399. doi: 10.1007/s10584-007-9249-0

[90] Lobell DB, Bänziger M, Magorokosho C, Vivek B. Nonlinear heat effects on African maize as evidenced by historical yield trials. Nat Clim Chang. 2011;1: 42 EP-. doi: 10.1038/nclimate1043

[91] Adhikari U, Nejadhashemi AP, Woznicki SA. Climate change and eastern Africa: A review of impact on major crops. Food Energy Secur. 2015;4: 110–132. doi: 10.1002/fes3.61

[92] Broberg MC, Högy P, Pleijel H. CO2-induced changes in wheat grain composition: Meta-analysis and response functions. Agronomy. 2017;7: 1–18. doi: 10.3390/agronomy7020032

[93] Lobell DB, Burke MB, Tebaldi C, Mastrandrea MD, Falcon WP, Naylor RL. Prioritizing climate change adaptation needs for food security in 2030. Science. 2008;319: 607–610. doi: 10.1126/science.1152339 PM– . 18239122

[94] Wallach D, Martre P, Liu B, Asseng S, Ewert F, Thorburn PJ, et al. Multimodel ensembles improve predictions of crop-environment-management interactions. Glob Chang Biol. 2018;24: 5072–5083. doi: 10.1111/gcb.14411 PM– . 30055118

[95] Araya A, Hoogenboom G, Luedeling E, Hadgu KM, Kisekka I, Martorano LG. Assessment of maize growth and yield using crop models under present and future climate in southwestern Ethiopia. Agric For Meteorol. 2015;214–215: 252–265. doi: 10.1016/j.agrformet.2015.08.259

[96] Lin Y, Feng Z, Wu W, Yang Y, Zhou Y, Xu C. Potential Impacts of Climate Change and Adaptation on Maize in Northeast China. Agron J. 2017;109: 1476. doi: 10.2134/agronj2016.05.0275

[97] Yin Y, Tang Q, Liu X. A multi-model analysis of change in potential yield of major crops in China under climate change. Earth Syst Dyn. 2015;6: 45–59. doi: 10.5194/esd-6-45-2015

[98] Li T, Hasegawa T, Yin X, Zhu Y, Boote K, Adam M, et al. Uncertainties in predicting rice yield by current crop models under a wide range of climatic conditions. Glob Chang Biol. 2015;21: 1328–1341. doi: 10.1111/gcb.12758 25294087

[99] Folberth C, Elliott J, Müller C, Balkovič J, Chryssanthacopoulos J, Izaurralde RC, et al. Parameterization-induced uncertainties and impacts of crop management harmonization in a global gridded crop model ensemble. PLoS ONE. 2019. doi: 10.1371/journal.pone.0221862 31525247 PMC6746385

[100] Kröbel R, Sun Q, Ingwersen J, Chen X, Zhang F, Müller T, et al. Modelling water dynamics with DNDC and DAISY in a soil of the North China Plain: A comparative study. Environ Model Softw. 2010;25: 583–601. doi: 10.1016/j.envsoft.2009.09.003

[101] Kersebaum KC, Boote KJ, Jorgenson JS, Nendel C, Bindi M, Frühauf C, et al. Analysis and classification of data sets for calibration and validation of agro-ecosystem models. Environ Model Softw. 2015;72: 402–417. doi: 10.1016/j.envsoft.2015.05.009

