# Supplementary figures and images for: Climate change impact on wheat and maize growth in Ethiopia: A multi-model uncertainty analysis
Source: PLoS One. 2022 Jan 21;17(1):e0262951. doi: 10.1371/journal.pone.0262951 (PMC8782302; doi:10.1371/journal.pone.0262951)

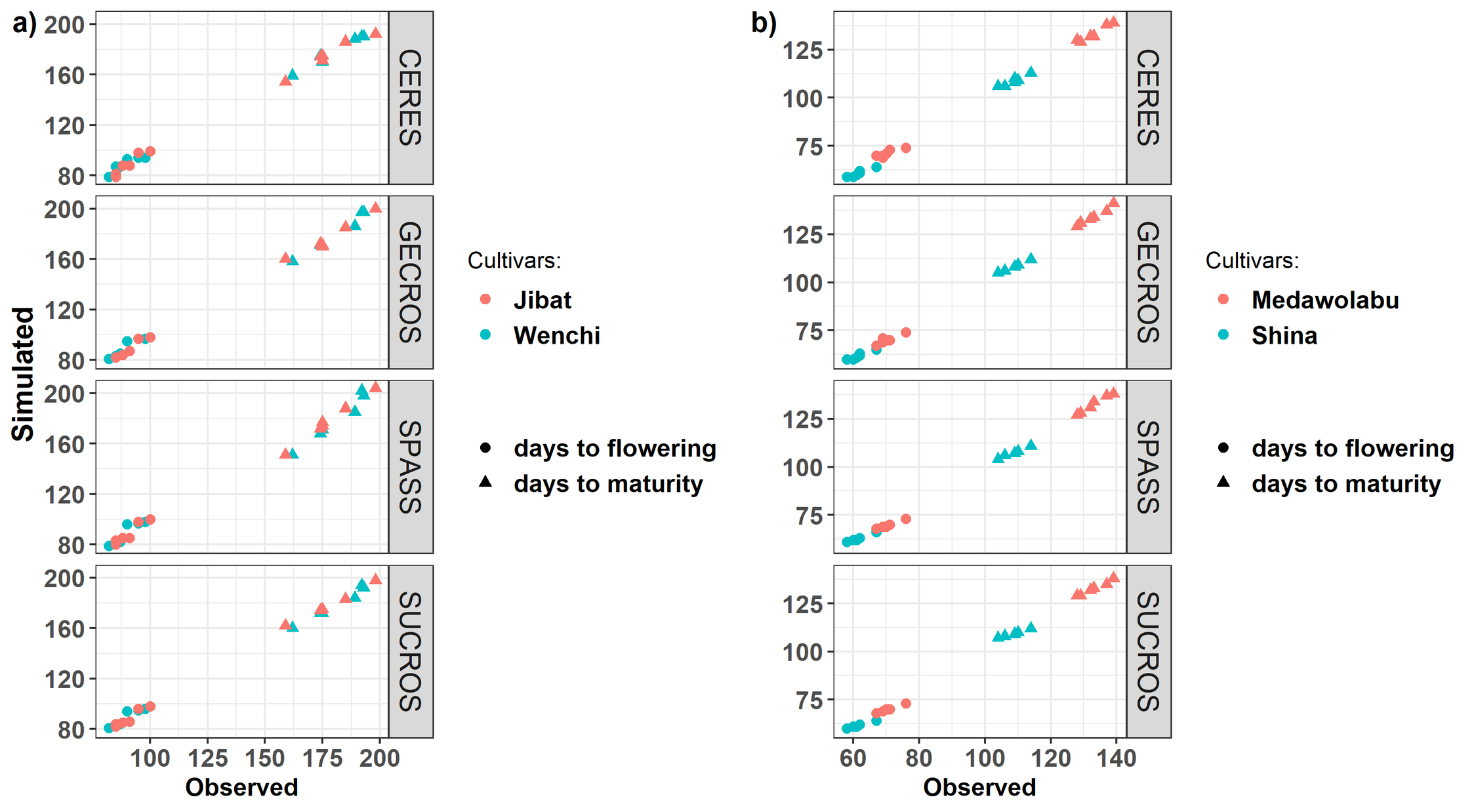

Supplement: S1 Fig — Scatter plot between model simulation and observation for days to flowering and days to maturity for the four plant growth submodels for maize (a) and wheat (b) cultivars. (TIF) [file pone.0262951.s001.tif]

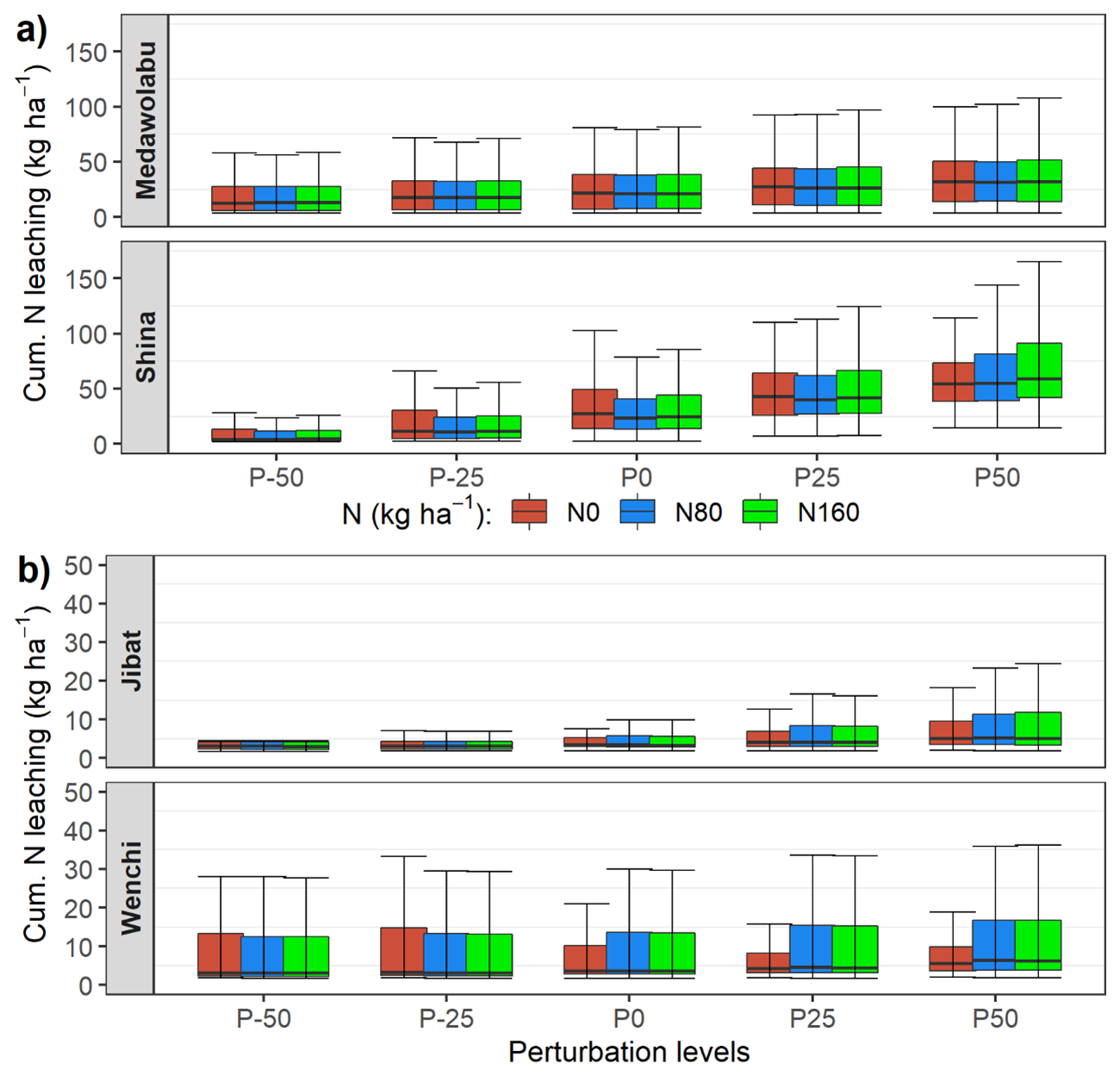

Supplement: S2 Fig — Effects of precipitation perturbations relative to the baseline climate on cumulative N leaching for (a) wheat and (b) maize cultivars. (TIF) [file pone.0262951.s002.tif]
