# Supplementary material for: Climate change impact on wheat and maize growth in Ethiopia: A multi-model uncertainty analysis
Source: PLoS One. 2022 Jan 21;17(1):e0262951. doi: 10.1371/journal.pone.0262951 (PMC8782302; doi:10.1371/journal.pone.0262951)
Supplement: S1 Table — (DOCX) [file pone.0262951.s003.docx]

| Model acronym | Model, Country | Reference |
| --- | --- | --- |
| CanESM2 | Second Generation Canadian Earth System Model, Canada | [1] |
| CNRM-CM5 | Centre National de Recherches Meteorologiques Coupled Global Climate Model, version 5, France | [2] |
| CSIRO-Mk3-6-0 | Commonwealth Scientific and Industrial Research Organization Mark, version 3.6.0, Australia | [3] |
| EC-EARTH | European community Earth-System Model, Netherlands/Ireland | [4] |
| IPSL-CM5A-MR | L’Institut Pierre-Simon Laplace Coupled Model, version 5A, coupled with NEMO, mid resolution, France | [5] |
| MIROC5 | Model for Interdisciplinary Research on Climate, version 5, Japan | [6] |
| HadGEM2-ES | Hadley Centre Global Environment Model, version 2–Earth System, United Kingdom | [7] |
| MPI-ESM-LR | Max Planck Institute Earth System Model, low resolution, Germany | [8] |
| NorESM1-M | Norwegian Earth System Model, version 1 (intermediate resolution) , Norway | [9] |
| GFDL-ESM2M | Geophysical Fluid Dynamics Laboratory Earth System Model with Modular Ocean Model 4 (MOM4) component, United States | [10,11] |

# Reference

1. Chylek P, Li J, Dubey MK, Wang M, Lesins G. Observed and model simulated 20th century Arctic temperature variability: Canadian Earth System Model CanESM2. Atmos Chem Phys Discuss. 2011;11: 22893–22907. doi:10.5194/acpd-11-22893-2011

2. Voldoire A, Sanchez-Gomez E, Salas y Mélia D, Decharme B, Cassou C, Sénési S, et al. The CNRM-CM5.1 global climate model: Description and basic evaluation. Clim Dyn. 2013;40: 2091–2121. doi:10.1007/s00382-011-1259-y

3. Collier MA, Jeffrey SJ, Rotstayn LD, Wong KKH, Dravitzki SM, Moeseneder C, et al. The CSIRO-Mk3.6.0 Atmosphere-Ocean GCM: Participation in CMIP5 and data publication. MODSIM 2011 - 19th Int Congr Model Simul - Sustain Our Futur Underst Living with Uncertain. 2011; 2691–2697.

4. Hazeleger W, Severijns C, Semmler T, Ştefănescu S, Yang S, Wang X, et al. EC-Earth: A Seamless Earth-System Prediction Approach in Action. Bull Am Meteorol Soc. 2010;91: 1357–1364. doi:10.1175/2010BAMS2877.1

5. Dufresne J-L, Foujols M-A, Denvil S, Caubel A, Marti O, Aumont O, et al. Climate change projections using the IPSL-CM5 Earth System Model: From CMIP3 to CMIP5. Clim Dyn. 2013;40: 2123–2165. doi:10.1007/s00382-012-1636-1

6. Watanabe M, Suzuki T, O’ishi R, Komuro Y, Watanabe S, Emori S, et al. Improved Climate Simulation by MIROC5: Mean States, Variability, and Climate Sensitivity. J Clim. 2010;23: 6312–6335. doi:10.1175/2010JCLI3679.1

7. Jones CD, Hughes JK, Bellouin N, Hardiman SC, Jones GS, Knight J, et al. The HadGEM2-ES implementation of CMIP5 centennial simulations. Geosci Model Dev. 2011;4: 543–570. doi:10.5194/gmd-4-543-2011

8. Giorgetta M, Jungclaus J, Reick C, Legutke S, Brovkin V, Crueger T, et al. CMIP5 simulations of the Max Planck Institute for Meteorology (MPI-M) based on the MPI-ESM-LR model: The rcp45 experiment, served by ESGF. World Data Center for Climate (WDCC) at DKRZ; 2012. doi:10.1594/WDCC/CMIP5.MXELR4

9. Bentsen M, Bethke I, Debernard JB, Iversen T, Kirkevåg A, Seland Ø, et al. The Norwegian Earth System Model, NorESM1-M -- Part 1: Description and basic evaluation of the physical climate. Geosci Model Dev. 2013;6: 687–720. doi:10.5194/gmd-6-687-2013

10. Dunne JP, John JG, Adcroft AJ, Griffies SM, Hallberg RW, Shevliakova E, et al. GFDL’s ESM2 Global Coupled Climate–Carbon Earth System Models. Part I: Physical Formulation and Baseline Simulation Characteristics. J Clim. 2012;25: 6646–6665. doi:10.1175/JCLI-D-11-00560.1

11. Dunne JP, John JG, Shevliakova E, Stouffer RJ, Krasting JP, Malyshev SL, et al. GFDL’s ESM2 Global Coupled Climate--Carbon Earth System Models. Part II: Carbon System Formulation and Baseline Simulation Characteristics*. J Clim. 2013;26: 2247–2267. doi:10.1175/JCLI-D-12-00150.1
