# Supplementary material for: Climate change impact on wheat and maize growth in Ethiopia: A multi-model uncertainty analysis
Source: PLoS One. 2022 Jan 21;17(1):e0262951. doi: 10.1371/journal.pone.0262951 (PMC8782302; doi:10.1371/journal.pone.0262951)
Supplement: S2 Table — (DOCX) [file pone.0262951.s004.docx]

| Parameter | Description | Unit |
| --- | --- | --- |
|  | **CERES** |  |
| PHINT | Phyllochrone interval | - |
| TMAXDEV | Maximum temperature for development | °C |
| TOPTDEV | Optimum temperatur for development | °C |
| TBASEDEV | Minimum temperature for development | °C |
| P1 | Thermal Time from emergence-to terminal spikelet ((phase 1) | °C d |
| P4 | Thermal Time from end of pre-anthesis ear growth - begin of grain filling (phase 4) | °C d |
| P5 | Thermal Time for grain filling (phase 5) | °C d |
| G1 | Number of grains per stem weight at anthesis | #/g |
| G2 | Maximum grain filling rate | g grain^-1^ d^-1^ |
| REXT | Maximum root extension rate | cm d^-1^ |
|  | **GECROS** |  |
| TBD | Base temperature for phenological development | °C |
| TOD | Optimum temperature for phenological development | °C |
| MTDV | Minimum thermal days of vegetative phase | d |
| MTDR | Minimum thermal days of reproductive phase | d |
| NUPTX | Maximum crop nitrogen uptake | g(N) m^-2^ d^-1^ |
| SLNMIN | Minimum (base) specific leaf nitrogen for photosynthesis | g(N) m^-2^ |
| LNCI | Initial leaf nitrogen concentration | g(N) g-1 |
| SLA0 | Specific leaf area | m^2^(leaf) g^-1^ |
| SEEDW | Seed weight | g seed^-1^ |
| SEEDNC | Standard seed (storage organ) nitrogen concentration | g(N) g^-1^ |
|  | **SPASS** |  |
| PDD1 | Physiological development days from emergence to anthesis | d |
| PDD2 | Physiological development days from anthesis to maturity | d |
| TMINPS | Minimum temperature of photosynthesis | °C |
| TOPTPS | Optimum temperature of photosynthesis | °C |
| TMAXPS | Maximum temperature of photosynthesis | °C |
| G1 | Number of grains per plant | # |
| SPCLW | Specific leaf weight | kg (DW) ha (leaf)^-1^ |
| G2 | Maximum grain filling rate | g grain^-1^ d^-1^ |
| PMAX | Gross photosynthesis rate at light saturation (at CO_2_ 340ppm) | kg (CO_2_) ha (leaf)^-1^ h^-1^ |
| LUE | Light use efficiency (at low light) | kg (CO_2_) ha (leaf)^-1^ h^-1^/(W m^-2^) |
|  | **SUCROS** |  |
| TBASE1 | Base temperature for phenological development during vegetative growth period | °C |
| TSUM1 | Temperature sum of vegetative growth phase | °C d |
| TBASE2 | Base temperature for phenological development during generative growth period | °C |
| TSUM2 | Temperature sum of generative growth phase | °C d |
| SPCLW | Specific leaf weight | kg (DW) ha (leaf)^-1^ |
| PMAX | Gross photosynthesis rate at light saturation (at CO_2_ 340ppm) | kg (CO_2_) ha (leaf)^-1^ h^-1^ |
| LA0 | Initial leaf area | m^2^/plant*10000 |
| G1 | Number of grains per plant | # |
| REXT | Maximum root extension rate | cm d^-1^ |
