# Supplementary material for: Climate change impact on wheat and maize growth in Ethiopia: A multi-model uncertainty analysis
Source: PLoS One. 2022 Jan 21;17(1):e0262951. doi: 10.1371/journal.pone.0262951 (PMC8782302; doi:10.1371/journal.pone.0262951)
Supplement: S3 Table — (DOCX) [file pone.0262951.s005.docx]

| Managment | Type | Maize^*^ |  | Wheat^+^ |  |
| --- | --- | --- | --- | --- | --- |
|  |  | Jibat | Wenchi | Medawolabu | Shina |
| Total applied N fertilizer (kg ha^-1^) | DAP (applied at planting) | 150 | 150 | 150 | 150 |
|  | UREA | 200 | 200 | 150 | 150 |
| Seed density (#/m^2^) |  | 7 | 7 | 300 | 250 |
| Row spacing (cm) |  | 75 | 75 | 12.5 | 12.5 |

^*^UREA was applied half at knee height and half at flag leaves emergency

^+^UREA half at planting and half at knee height
