# Supplementary material for: Climate change impact on wheat and maize growth in Ethiopia: A multi-model uncertainty analysis
Source: PLoS One. 2022 Jan 21;17(1):e0262951. doi: 10.1371/journal.pone.0262951 (PMC8782302; doi:10.1371/journal.pone.0262951)
Supplement: S4 Table — It lists the performance of the four of plant growth submodels for each crop cultivar. (DOCX) [file pone.0262951.s006.docx]

| **Plant growth**  **submodels** | **Days to flowering** | | | | **Days to maturity** | | | |
| --- | --- | --- | --- | --- | --- | --- | --- | --- |
|  | NSE | r | RMSE | PBIAS | NSE | r | RMSE | PBIAS |
| **Medawolabu (wheat)** | | | | | | | | |
| CERES | 0.599 | 0.860 | 1.780 | 1.200 | 0.936 | 0.971 | 1.000 | 0.300 |
| GECROS | 0.810 | 0.917 | 1.225 | -0.200 | 0.883 | 0.985 | 1.354 | 0.900 |
| SPASS | 0.746 | 0.990 | 1.414 | -0.900 | 0.947 | 0.984 | 0.913 | -0.400 |
| SUCROS | 0.768 | 0.994 | 1.354 | -0.700 | 0.936 | 0.988 | 1.000 | -0.300 |
| **Shina (wheat)** | | | | | | | | |
| CERES | 0.713 | 0.947 | 1.472 | -1.400 | 0.865 | 0.946 | 1.155 | 0.000 |
| GECROS | 0.801 | 0.947 | 1.225 | 0.300 | 0.865 | 0.995 | 1.155 | -0.600 |
| SPASS | 0.625 | 0.990 | 1.683 | 1.900 | 0.646 | 0.986 | 1.871 | -1.400 |
| SUCROS | 0.691 | 0.989 | 1.528 | 0.000 | 0.713 | 0.989 | 1.683 | 0.500 |
| **Wenchi (Maize)** | | | | | | | | |
| CERES | 0.790 | 0.896 | 2.550 | -0.600 | 0.937 | 0.987 | 2.858 | -1.200 |
| GECROS | 0.790 | 0.935 | 2.550 | 0.200 | 0.871 | 0.984 | 4.082 | -0.600 |
| SPASS | 0.580 | 0.919 | 3.606 | -0.400 | 0.595 | 0.979 | 7.234 | -0.900 |
| SUCROS | 0.833 | 0.932 | 2.273 | -0.600 | 0.939 | 0.986 | 2.799 | -1.000 |
| **Jibat (Maize)** | | | | | | | | |
| CERES | 0.600 | 0.954 | 3.440 | -2.000 | 0.908 | 0.974 | 3.606 | -1.300 |
| GECROS | 0.673 | 0.964 | 3.109 | -2.600 | 0.941 | 0.982 | 2.887 | -0.800 |
| SPASS | 0.532 | 0.948 | 3.719 | -2.400 | 0.860 | 0.996 | 4.435 | 0.000 |
| SUCROS | 0.724 | 0.956 | 2.858 | -2.400 | 0.985 | 0.995 | 1.472 | 0.100 |

Pearson correlation coefficient (r), Root Mean Square Error (RMSE), the percent bias (PBIAS) and Nash-Sutcliffe efficiency (NSE)
