# Supplementary material for: Climate change impact on wheat and maize growth in Ethiopia: A multi-model uncertainty analysis
Source: PLoS One. 2022 Jan 21;17(1):e0262951. doi: 10.1371/journal.pone.0262951 (PMC8782302; doi:10.1371/journal.pone.0262951)
Supplement: S5 Table — It lists the performance of the factorial combination of plant growth x soil water flow x soil organic matter submodels and modules. (DOCX) [file pone.0262951.s007.docx]

| Plant growth  submodels | Soil water flow  submodels | SOM  submodels | **Grain yield** | | | |
| --- | --- | --- | --- | --- | --- | --- |
|  |  |  | NSE | r | RMSE | PBIAS |
| **Medawolabu (wheat)** | | | | | | |
| CERES | Richards | SoilN | 0.75 | 0.89 | 2.93 | 0.7 |
| CERES | Richards | DAISY | 0.68 | 0.84 | 3.31 | 1.2 |
| CERES | Capacity | SoilN | 0.41 | 0.87 | 4.51 | 0.6 |
| CERES | Capacity | DAISY | 0.35 | 0.86 | 4.73 | 0.1 |
| GECROS | Richards | SoilN | 0.22 | 0.92 | 5.19 | 0.2 |
| GECROS | Richards | DAISY | 0.22 | 0.92 | 5.19 | 0.1 |
| GECROS | Capacity | SoilN | 0.21 | 0.91 | 5.23 | -1.3 |
| GECROS | Capacity | DAISY | 0.21 | 0.91 | 5.23 | -0.2 |
| SPASS | Richards | SoilN | 0.37 | 0.87 | 4.68 | 1.1 |
| SPASS | Richards | DAISY | 0.37 | 0.87 | 4.68 | 1.1 |
| SPASS | Capacity | SoilN | 0.4 | 0.72 | 4.53 | 1.9 |
| SPASS | Capacity | DAISY | 0.39 | 0.7 | 4.57 | 1.7 |
| SUCROS | Richards | SoilN | 0.44 | 0.88 | 4.38 | 0 |
| SUCROS | Richards | DAISY | 0.44 | 0.88 | 4.38 | 0 |
| SUCROS | Capacity | SoilN | 0.44 | 0.88 | 4.39 | 0 |
| SUCROS | Capacity | DAISY | 0.44 | 0.88 | 4.39 | 0 |
| **Shina (wheat)** | | | | | | |
| CERES | Richards | SoilN | 0.75 | 0.89 | 1.24 | 0.2 |
| CERES | Richards | DAISY | 0.74 | 0.89 | 1.27 | 0.4 |
| CERES | Capacity | SoilN | 0.52 | 0.75 | 1.72 | 1.1 |
| CERES | Capacity | DAISY | 0.37 | 0.61 | 1.97 | 0.4 |
| GECROS | Richards | SoilN | 0.28 | 0.54 | 2.1 | 0.4 |
| GECROS | Richards | DAISY | 0.29 | 0.56 | 2.08 | 0.9 |
| GECROS | Capacity | SoilN | 0.28 | 0.55 | 2.1 | 0.7 |
| GECROS | Capacity | DAISY | 0.28 | 0.54 | 2.11 | 0.7 |
| SPASS | Richards | SoilN | 0.65 | 0.83 | 1.47 | -0.9 |
| SPASS | Richards | DAISY | 0.65 | 0.82 | 1.46 | -0.3 |
| SPASS | Capacity | SoilN | 0.48 | 0.76 | 1.79 | -1.4 |
| SPASS | Capacity | DAISY | 0.41 | 0.75 | 1.9 | -1.9 |
| SUCROS | Richards | SoilN | 0.42 | 0.66 | 1.89 | 0.2 |
| SUCROS | Richards | DAISY | 0.41 | 0.65 | 1.91 | 0.2 |
| SUCROS | Capacity | SoilN | 0.44 | 0.68 | 1.85 | 0.5 |
| SUCROS | Capacity | DAISY | 0.44 | 0.68 | 1.85 | 0.5 |
| **Wenchi (Maize)** | | | | | | |
| CERES | Richards | SoilN | 0.75 | 0.89 | 5.27 | -1 |
| CERES | Richards | DAISY | 0.72 | 0.87 | 5.55 | 0.2 |
| CERES | Capacity | SoilN | 0.43 | 0.66 | 7.98 | 0.4 |
| CERES | Capacity | DAISY | 0.52 | 0.72 | 7.32 | 0.7 |
| GECROS | Richards | SoilN | 0.89 | 0.97 | 3.44 | -0.2 |
| GECROS | Richards | DAISY | 0.92 | 0.98 | 3 | 0.7 |
| GECROS | Capacity | SoilN | 0.37 | 0.81 | 8.34 | 1.8 |
| GECROS | Capacity | DAISY | 0.18 | 0.5 | 9.57 | 0.1 |
| SPASS | Richards | SoilN | 0.4 | 0.86 | 8.16 | 0.1 |
| SPASS | Richards | DAISY | 0.25 | 0.7 | 9.14 | -0.1 |
| SPASS | Capacity | SoilN | 0.17 | 0.42 | 9.59 | 0.7 |
| SPASS | Capacity | DAISY | 0.21 | 0.6 | 9.37 | -0.4 |
| SUCROS | Richards | SoilN | 0.93 | 0.96 | 2.88 | -0.2 |
| SUCROS | Richards | DAISY | 0.95 | 0.98 | 2.3 | -0.5 |
| SUCROS | Capacity | SoilN | 0.84 | 0.97 | 4.27 | 0.2 |
| SUCROS | Capacity | DAISY | 0.84 | 0.97 | 4.27 | 0.2 |
| **Jibat (Maize)** | | | | | | |
| CERES | Richards | SoilN | 0.81 | 0.91 | 4.99 | -0.4 |
| CERES | Richards | DAISY | 0.81 | 0.91 | 5.06 | 0.1 |
| CERES | Capacity | SoilN | 0.45 | 0.69 | 8.6 | -0.8 |
| CERES | Capacity | DAISY | 0.44 | 0.68 | 8.69 | -0.9 |
| GECROS | Richards | SoilN | 0.94 | 0.97 | 2.77 | 0.1 |
| GECROS | Richards | DAISY | 0.93 | 0.97 | 2.95 | 0.2 |
| GECROS | Capacity | SoilN | 0.3 | 0.57 | 9.66 | 1.6 |
| GECROS | Capacity | DAISY | 0.25 | 0.52 | 10 | 0.8 |
| SPASS | Richards | SoilN | 0.79 | 0.89 | 5.29 | -0.9 |
| SPASS | Richards | DAISY | 0.63 | 0.95 | 7.09 | 0.4 |
| SPASS | Capacity | SoilN | 0.28 | 0.82 | 9.85 | -0.2 |
| SPASS | Capacity | DAISY | 0.29 | 0.68 | 9.78 | 0.5 |
| SUCROS | Richards | SoilN | 0.73 | 0.85 | 6.07 | -0.2 |
| SUCROS | Richards | DAISY | 0.67 | 0.82 | 6.68 | 0 |
| SUCROS | Capacity | SoilN | 0.89 | 0.95 | 3.76 | 0.9 |
| SUCROS | Capacity | DAISY | 0.89 | 0.95 | 3.75 | 0.9 |

Pearson correlation coefficient (r), Root Mean Square Error (RMSE), the percent bias (PBIAS) and Nash-Sutcliffe efficiency (NSE)
