# Supplementary material for: Climate change impact on wheat and maize growth in Ethiopia: A multi-model uncertainty analysis
Source: PLoS One. 2022 Jan 21;17(1):e0262951. doi: 10.1371/journal.pone.0262951 (PMC8782302; doi:10.1371/journal.pone.0262951)
Supplement: S6 Table — (DOCX) [file pone.0262951.s008.docx]

**S6 Table. Analysis of variance (ANOVA) table for the response of grain yield to N fertilizer (N), CO2, temperature (T), and precipitation (P) treatments**

| [1] Medawolabu (Wheat) | | | | | |
| --- | --- | --- | --- | --- | --- |
|  | Df | Sum Sq | Mean Sq | F value | Pr(>F) |
| T | 3 | 21792 | 7264 | 9666.21 | <2e-16 *** |
| P | 4 | 2074 | 519 | 690.1 | <2e-16 *** |
| CO2 | 4 | 5012 | 1253 | 1667.3 | <2e-16 *** |
| N | 2 | 18779 | 9390 | 12494.89 | <2e-16 *** |
| T:N | 6 | 1698 | 283 | 376.57 | <2e-16 *** |
| P:N | 8 | 1423 | 178 | 236.64 | <2e-16 *** |
| CO2:N | 8 | 556 | 69 | 92.43 | <2e-16 *** |
| [2] Shina (Wheat) | | | | | |
|  | Df | Sum Sq | Mean Sq | F value | Pr(>F) |
| T | 3 | 13455 | 4485 | 9715.3 | <2e-16 *** |
| P | 4 | 2378 | 595 | 1287.8 | <2e-16 *** |
| CO2 | 4 | 2946 | 737 | 1595.5 | <2e-16 *** |
| N | 2 | 41142 | 20571 | 44560.8 | <2e-16 *** |
| T:N | 6 | 2666 | 444 | 962.5 | <2e-16 *** |
| P:N | 8 | 916 | 115 | 248.1 | <2e-16 *** |
| CO2:N | 8 | 657 | 82 | 178.0 | <2e-16 *** |
| [3] Jibat (Maize) | | | | | |
|  | Df | Sum Sq | Mean Sq | F value | Pr(>F) |
| T | 3 | 923 | 308 | 111.272 | < 2e-16 *** |
| P | 4 | 223 | 56 | 20.152 | < 2e-16 *** |
| CO2 | 4 | 206 | 51 | 18.609 | 2.72e-15 *** |
| N | 2 | 61202 | 30601 | 11067.81 | < 2e-16 *** |
| T:N | 6 | 88 | 15 | 5.296 | 1.81e-05 *** |
| P:N | 8 | 267 | 33 | 12.073 | < 2e-16 *** |
| CO2:N | 8 | 6 | 1 | 0.277 | 0.974 |
| [4] Wenchi (Maize) | | | | | |
|  | Df | Sum Sq | Mean Sq | F value | Pr(>F) |
| T | 3 | 740 | 247 | 85.945 | < 2e-16 *** |
| P | 4 | 327 | 82 | 28.488 | < 2e-16 *** |
| CO2 | 4 | 206 | 52 | 17.963 | 9.50e-15 *** |
| N | 2 | 81560 | 40780 | 14203.9 | < 2e-16 *** |
| T:N | 6 | 114 | 19 | 6.617 | 5.25e-07 *** |
| P:N | 8 | 325 | 41 | 14.156 | < 2e-16 *** |
| CO2:N | 8 | 11 | 1 | 0.465 | 0.882 |
| Signif. codes: 0 '***' 0.001 '**' 0.01 '*' 0.05 '.' 0.1 ' ' 1 | | | | | |
