# Supplementary material for: Climate change impact on wheat and maize growth in Ethiopia: A multi-model uncertainty analysis
Source: PLoS One. 2022 Jan 21;17(1):e0262951. doi: 10.1371/journal.pone.0262951 (PMC8782302; doi:10.1371/journal.pone.0262951)
Supplement: S1 Text — (DOCX) [file pone.0262951.s009.docx]

**Table S1 Response of grain yield to N fertilizer, CO2, temperature, and precipitation treatments**

**[1] "Yield response to N"**

Df Sum Sq Mean Sq F value Pr(>F)
 Cultivar 3 47272 15757 9003 <2e-16 ***
 N 4 16340 4085 2334 <2e-16 ***
 Residuals 19342 33854 2
 ---

[2] " Yield response to CO2"
 Df Sum Sq Mean Sq F value Pr(>F)
 Cultivar 3 112367 37456 16631.6 <2e-16 ***
 CO2 4 4735 1184 525.7 <2e-16 ***
 N 2 79134 39567 17569.2 <2e-16 ***
 Residuals 58040 130711 2
 ---

**[3] " Yield response to Temperature (T)"** Df Sum Sq Mean Sq F value Pr(>F)
 Cultivar 3 184947 61649 44255 <2e-16 ***
 T 3 8941 2980 2140 <2e-16 ***
 N 2 38250 19125 13729 <2e-16 ***
 Residuals 46431 64681 1

[4] " Yield response to Precipitation (P)"
 Df Sum Sq Mean Sq F value Pr(>F)
 Cultivar 3 144538 48179 25497.7 <2e-16 ***
 P 4 928 232 122.8 <2e-16 ***
 N 2 69564 34782 18407.7 <2e-16 ***
 Residuals 58040 109670 2
 ---
 Signif. codes: 0 '***' 0.001 '**' 0.01 '*' 0.05 '.' 0.1 ' ' 1
